# Element Specific *Versus* Integral Structural and Magnetic Properties of Co:ZnO and Gd:GaN Probed with Hard X-ray Absorption Spectroscopy

**DOI:** 10.3390/ma3063565

**Published:** 2010-06-07

**Authors:** Andreas Ney

**Affiliations:** Fakultät für Physik, Universität Duisburg-Essen, Lotharstr. 1, D-47057 Duisburg, Germany; E-Mail: andreas.ney@uni-due.de; Tel.: +49-203-379-2381; Fax: +49-203-379-2098

**Keywords:** dilute magnetic semiconductors, magnetic properties, X-ray absorption spectroscopy

## Abstract

Dilute magnetic semiconductors (DMS) are envisioned as sources of spin-polarized carriers for future semiconductor devices which simultaneously utilize spin and charge of the carriers. The hope of discovering a DMS with ferromagnetic order up to room temperature still motivates research on suitable DMS materials. Two candidate wide-band gap DMS are Gd:GaN and Co:ZnO. We have used hard X-ray absorption spectroscopy (XAS) and in particular X-ray linear dichroism (XLD) and X-ray magnetic circular dichroism (XMCD) to study both DMS materials with element specificity and compare these findings with results from integral SQUID magnetometry as well as electron paramagnetic resonance (EPR).

## 1. Introduction

The story of transistor-based integrated circuits for modern computing is a story of great success; oftentimes it is therefore called micro-electronic revolution. The proven concept for enhancing computational power by continuous miniaturization (*i.e.*, increasing the integration density) has been predicted to follow an exponential law [1], which is nowadays known as “Moore’s law". However, as any exponential growth, also the continuous miniaturization is approaching its fundamental limits, *i.e.*, the atomic scale. At all times novel materials have been at the forefront of keeping up the pace of the microelectronics industry. For example, novel low-*k* dielectrics based on hafnium oxide have replaced the silicon dioxide gate insulation in the current generation of microprocessors of the 45 nm generation. In parallel to the progress of the integrated circuits, also storage technology has followed its own exponential growth in data-density, sometimes even outpacing Moore’s law. In this area the spin rather than the charge of the electron is utilized in the sense that ferromagnetic materials are used to represent the information by its magnetization direction in a non-volatile fashion. This principle has been used in any hard disk drive or magnetic tape storage from the nineteen-fifties until today.

Facing the fundamental limitations of the growth in integration density, novel computational concepts came into the focus. A variety of new concepts were proposed in the last years, e.g., upgrading the functionality of the common transistor by utilizing also the spin information of the electron [2,3,4] or replacing the silicon-based transistors by nanoscale switches made of carbon nanotubes [5,6] or molecules [7]. These concepts have been intensely pursued over the last decade by researchers in academia as well as industry. Especially the former proposal to utilize the electron’s spin inside a semiconductor device, nowadays known as spin-electronics or “spintronics" [4], comprises novel magneto-electric effects which are nowadays used in every day’s microelectronic devices. In particular, the enormous growth of the storage density of hard-disk drives is owing to the discovery of the giant magneto-resistance (GMR) effect [8,9], which enabled the reliable electronic read-out of magnetic bits with drastically decreased size. GMR-elements consist of two magnetic layers which are separated by a non-magnetic spacer, or alternatively, a tunnel barrier (so-called tunneling magnetoresistance, TMR [10]). The resistance of the GMR/TMR device depends strongly on the relative orientation of the magnetization of the two magnetic layers. Such GMR/TMR elements are the functional heart of magnetic random access memory (MRAM) [11].

Besides the enormous technological impact of the GMR/TMR element as first “real-working" spintronics device in modern storage technology, another aspect of MRAM-like structures came into focus. Recently, novel concepts for magnetic logic were proposed using MRAM-like elements [12,13,14]. Direct programmability of logic gates, *i.e.*, the change of the logic function of an individual gate, is inherently impossible for transistor-based logic where the logic function is determined by the interconnect wiring. The intrinsic non-volatility of MRAM-like structures allows run-time configurable logic gates based on a single MRAM element [14]. However, this concept can be easily transferred to other spintronic devices. The basic functional concept is based on a four-level system (like two ideal magnetic layers) where an electronic effect (like resistance) only depends on the relative orientation of the two sub-systems (like parallel or antiparallel alignment of the two magnetizations). It is therefore obvious, that the use of the spin degree of freedom can offer a variety of opportunities to increase the performance of logic devices by enabling programmability and/or storage capabilities.

Integrating the spin degree of freedom into semiconductor devices has been usually done by creating a spin population inside a semiconductor by spin-injection; the detection of successful spin injection is to measure the polarization of the injected carriers optically via the circular polarization of light emitted from a quantum well within the semiconductor. This structure is often referred to as a spin-LED [3,15]. Small values of spin injection at room-temperature have been detected with such a device having injectors based on Fe layers and a naturally formed Schottky barrier [16]. Significantly higher spin injection efficiencies have been found by replacing the Schottky-barrier with an oxide tunnel barrier with values as high as 30% at room temperature [17,18]. More recently, also electrical detection of the spin polarization has successfully been demonstrated [19]. An elegant way to circumvent possible issues arising from, e. g., non-ideal interfaces between ferromagnets and semiconductors would be to base spintronic devices directly on ferromagnetic semiconductors. This would allow both the utilization of the intrinsic spin polarization as well as the semiconducting properties of the material at the same time.

## 2. Dilute Magnetic Semiconductors (DMS)

The discovery of ferromagnetic semiconductors dates back to the nineteen-sixties, when it was shown that EuO is a ferromagnetic semiconductor [20]. Recently, integration of EuO with silicon and GaN has been demonstrated making it a valuable material for proof-of-principle spintronic demonstrators [21]. However, the Curie temperature $T_{C}$ of EuO is well-below room-temperature (RT). Therefore, EuO cannot be considered to be useful for practical applications.

On the other hand, introducing a dopant into a solid, especially a semiconductor, is a well-known concept of manipulating its physical properties. It is therefore not surprising that the introduction of paramagnetic dopant atoms such as Mn into semiconductors has been a research field of considerable interest [22]. Most of these dilute magnetic semiconductors (DMS) were found to be paramagnetic (PM) and next cation-neighbor magnetic interactions have been intensely studied [23]. From the viewpoint of spintronic applications the turning-point was the demonstration that it it possible to achieve ferromagnetic ordering with $T_{C}$ as high as 60 K in GaAs doped with 3.5% of Mn [24]. Two additional benefits of DMS materials should be mentioned here, namely the possibility to control the magnetic order via the carrier concentration which can be easily varied by an gate electrode [25], and the possibility to switch the magnetization by the spin-torque effect with much lower current densities compared to conventional ferromagnetic metals [26]. The most severe drawback, however, is that up to now the $T_{C}$ of Mn-doped GaAs could not be increased above ∼180 K [27]. The Zener model could be employed to describe the magnetic properties of Mn-doped GaAs rather successfully and it further predicted, that it may become feasible to achieve $T_{C}$’s above RT for ZnO- and GaN-based DMS [28]. This prediction, although exclusively based on Mn-doping and on a very high hole concentration of >10${}^{20}$/cm${}^{3}$, sparked intense research efforts to synthesize DMS materials based on ZnO and GaN. Around the same time, also ZnO doped with other 3d transition metals was predicted to have a potential towards RT ferromagnetism [29]. One year later first claims of ferromagnetism at RT were made by experimentalists for Mn-doped GaN [30] and Co-doped ZnO [31]. The existence of RT ferromagnetism in Mn-doped GaN was quickly attributed to Mn-rich nanocrystallites [32] whereas the homogeneous, crystallographically excellent material was shown to behave like a spin-glass [33] or a ferromagnet with very low $T_{C}$ [34]. In parallel, doping GaN with Gd was stirring the interest of the materials community due to claims of RT ferromagnetism [35] even present at very dilute doping levels [36].

This review focuses on two of the DMS materials, for which claims of RT ferromagnetism exist throughout the literature until today. One compound is Co-doped ZnO (Zn${}_{1-x}$Co${}_{x}$O with *x* from 0.05 to 0.15; in short “Co:ZnO" in the following), which is heavily studied and debated throughout the literature over the last nine years. The other is Gd-doped GaN (Ga${}_{1-x}$Gd${}_{x}$N with *x* from 0.005 to 0.03, in short “Gd:GaN") where in particular the claims of colossal magnetic moments at low Gd concentrations [36] attracted a great deal of interest and controversy. The approach of this review is to study both wide-band gap DMS materials using a comprehensive set of structural and magnetic characterization techniques. Special attention will be paid to base the magnetic characterization on more than one experimental method to substantiate or disprove the existence of ferromagnetism in the materials in question. This is complemented by a synchrotron-based approach to study the structural properties with element specificity and finally to correlate the magnetic with the structural properties. Especially the latter aspect requires that a specific sample specimen is available at the respective beamlines of the synchrotron, therefore naturally limiting the number of studied samples. This review is therefore rather limited in the number and origin of the various specimens in question; however, the studied specimen were carefully selected and are representative for the DMS material under investigation. In addition, where available, the presented findings will be compared with the literature. Before the experimental techniques will be discussed in greater detail, a brief overview on the relevant literature available for the two DMS materials, Co:ZnO and Gd:GaN shall be given.

### 2.1. The Controversy about Co:ZnO DMS

#### 2.1.1. Experimental Work

Already in the first publication claiming RT ferromagnetism in Co:ZnO it was stated that “the reproducibility of the method was poor (less than 10%)" [31]. The situation with disparate claims of the existence or non-existence of RT ferromagnetism in Co:ZnO did not significantly improve throughout the last nine years, which was recently captured in two comprehensive review articles about thin-film oxides also comprising sections dealing with Co:ZnO [37,38], contrasted by a review on dilute magnetic oxides [39]. To provide the reader with a flavor about the recent controversy, a brief and subjective excerpt of the wealth of relevant literature will be given in the following.

Regarding the existence of RT ferromagnetism in Co:ZnO the reports range from no observation of ferromagnetism [40,41,42,43,44,45,46,47,48,49,50] over ferromagnetism with small effective moments per Co [51,52,53] to RT ferromagnetism with large Co moments approaching 3 $\mu_{B}$/atom [54,55,56,57,58,59]. A few claims of even higher effective magnetic moments can be found [60,61]. In particular, several groups have detected ferromagnetic behavior only by integral superconducting quantum interference device (SQUID) magnetometry, although element-specific synchrotron techniques fail to establish its presence [62,63]. This has been attributed to the important role played by defects for the ferromagnetic order [63]. Defects were also held responsible for the observed magnetic order which was claimed to be induced via the coalescence of so-called bound magnetic polarons formed by defects such as oxygen vacancies [64]. However, the experimental evidence based on integral magnetic data was criticized later-on [37]. More recently, the role of defects was also studied by analyzing published structural information based on X-ray diffraction (XRD) for pure and Mn-doped ZnO [65] inferring that a grain-boundary foam in ZnO may be the source of ferromagnetism. Recently, RT ferromagnetism induced by ball-milling of Al:ZnO has been discussed in terms of defects as well, since besides the Al no other dopant was nominally present in the ZnO [66]. On the other hand, a powder of Co:ZnO nanorods intrinsically containing many grain boundaries was found to be paramagnetic both by means of SQUID and synchrotron-based magnetometry [67].

The situation becomes even more intricate, if the role of the carriers which are generally *n*-type in ZnO is explicitly mentioned. Recently, two ferromagnetic regimes separated by a non-ferromagnetic one were suggested as a function of the *n*-type carrier concentration [57] supporting earlier findings highlighting the role of the carriers [68]. This being also in-line with reports of RT-ferromagnetism in ball-milled Al:ZnO [66]. On the other hand, the lack of ferromagnetism in *n*-type Co:ZnO was reported as well [47,50]. One important aspect in sorting out this puzzling situation may be to first discuss the structural details of the samples. The possibility that magnetic nanoclusters (which can also stem from contaminations in high purity powders, in cases where bulk-like quantities are used as specimen) play an important role in accounting for the observed magnetic behavior is under discussion over the last few years [69,70]. In particular, it had been shown that Al-codoping of Co:ZnO, which is used to increase the *n*-type carrier concentration, also promotes the onset of phase separation [71]. For this observation slow, careful XRD scans were necessary to detect Co metal inclusions which were missed under conventional measurement conditions. A comparably careful XRD experiment has recently led to identical conclusions and in addition, such nanoclusters could be imaged by cross-sectional transmission electron microscopy (TEM) [48,72]. Also synchrotron-based XRD can be used to detect the formation of metallic Co precipitations even in Co-implanted ZnO with low Co concentrations [73]. It is noteworthy, that recently also the combination of TEM and magnetic resonance measurements found phase separated nanocolumns responsible for RT ferromagnetism in the Co:ZnO system [74]. Finally, typical means aiming at the manipulation of the carrier concentration such as annealing in Zn vapor, was recently shown to lead to metallic ZnCo precipitations found by careful experiments using depth-profiling X-ray photoelectron spectroscopy (XPS) [75], which were corroborated by synchrotron-based techniques [72,76]. Ruling out phase separation is thus of utmost importance before RT ferromagnetism can be claimed.

#### 2.1.2. Theoretical Work

The theoretical prediction of room temperature ferromagnetism in transition metal doped ZnO was controversial from the beginning as well. Whereas ferromagnetism above RT was predicted based on the Zener model, 5% of Mn doping, and a high hole concentration [28] calculations based on the Korringa-Kohn-Rostoker (KKR) Green’s function method based on the local density approximation (LDA) found antiferromagnetism for Mn doping whereas Co doping should lead to ferromagnetism [29]. Since these initial predictions have been made, several theoretical calculations based on density functional theory (DFT) have been presented to explore the ferromagnetic ordering in Co:ZnO, e. g., [77,78,79,80,81]. In general, these calculations have indicated that insulating Co:ZnO is not ferromagnetic, and thus defects which add carriers to the system are necessary to stabilize the ferromagnetic phase. The nature of the defect varies with the specific calculation, with some predicting electron-mediated ferromagnetism [77,79] and others predicting hole mediation [78,80]; another calculation found that both electrons and holes could promote ferromagnetism in Co:ZnO [81].

One major drawback of these theoretical results is the well-known tendency of DFT calculations to significantly underestimate the bandgap of transition metal oxides, making accurate determination of the position of the Co and defect states within the ZnO band structure difficult or impossible [82]. In [82] several likely defects were explored as well and ferromagnetism was only found for the singly charged oxygen vacancy ($V^{+}$O), although this defect was predicted to be energetically unfavorable compared to *V*O and $V^{++}$O. Such DFT calculations generally predict half-metallic behavior for doped transition metal oxides, with the Fermi level crossing dopant-induced defect states in the middle of the host oxide bandgap [83]. Several more recent calculations utilizing modified methods like LDA+U [83] or pseudo-self-interaction-corrected LDA [84] have placed the occupied majority-spin Co 3*d* orbitals of *e* and $t_{2}$ symmetry within the ZnO valence band, and the occupied minority-spin *e* orbitals extending into the lower portion of the bandgap. The unoccupied minority-spin Co 3*d* $t_{2}$ orbitals were predicted to lie in the lower portion of the conduction band, and no levels were predicted at midgap. This picture is consistent with photoemission studies of Co:ZnO [69,85] which show Co:ZnO to be a semiconductor with additional states extending from the top of the valence band and no states observed at the Fermi level, a conclusion recently being confirmed by a combination of experiment and theory [86].

In summary, calculations predict that defect-free, insulating Co:ZnO is not ferromagnetic [82,83] whereas the role of *n*-type carriers remains under debate ranging from ferromagnetic coupling [79], over oscillatory with Co-Co distance [87] to antiferromagnetic coupling [50], which even increases with Co cluster size [88], where in this case “cluster" refers to Co on Zn lattice sites arranged in Co-rich areas embedded in a Co-poor ZnO matrix, a scenario which was used to explain weak ferromagnetic-like signatures by uncompensated magnetic moments at the surface of such clusters [89]. Finally, it was reported by theory that the formation of these types of Co clusters is energetically favorable [50,90]. It was even suggested that the variation of the magnetic moment on the carrier concentration inferred by experiments may be indirect because it can be a consequence of the variation in cluster size distributions that follows as a direct consequence of the chemical composition [50].

The above overview of both experimental and theoretical work indicates that the controversy about the magnetic properties of Co:ZnO may be caused—at least in parts—by the fact that it is experimentally very challenging to rule out the formation of secondary Co-containing phases which can account for ferromagnetic-like behavior. A meaningful structural and magnetic characterization is thus of utmost importance. One particular aim of this review is to shown how claims of RT ferromagnetism can be substantiated or disproved predominantly using synchrotron-based techniques, which have always played a central role in magnetism-related materials research [91].

### 2.2. Gd:GaN-RT Ferromagnetism with Colossal Moments?

Other than for the Co:ZnO DMS material, the available literature on Gd:GaN is less comprehensive and contains less controversy. The first claim of RT-ferromagnetism in Gd-doped GaN dates back to 2002 [35] and was detailed later-on [92]. Receiving little interest from the spintronic materials community in the beginning, rare-earth doping of GaN was primarily in the focus because of its importance in optoelectronics [93,94]. This has changed, when RT ferromagnetism was claimed to be present even at the very dilute doping level of the order of 10${}^{16}$/cm${}^{3}$ accompanied by effective magnetic moments of the order of 1000 $\mu_{B}$ per Gd atom [36]. These claims were accompanied by a comprehensive materials characterization to rule out eventual phase separation [95]. Later, these large effective moments per Gd atom were confirmed independently by another experimental group [96] and secondary phases were ruled out by synchrotron-based techniques [97]. Similar results, with even higher effective magnetic moments were also reported for Gd-ion-implanted hexagonal GaN [98]. On the other hand, in cases where Gd-ions were implanted into cubic GaN, only paramagnetism was found [99]. Besides the claims of colossal effective moments, the presence of RT-ferromagnetism was confirmed by other experimentalists up to Gd concentrations of 8.9% [100]. Special attention was paid to the correlation of RT-ferromagnetism with the preparation conditions [101,102], especially regarding the robustness of the RT-ferromagnetism with respect to defects [103]. The interrelation of RT-ferromagnetism with defects is also underlined by the existence of variable range hopping transport in ferromagnetic Gd:GaN [104]. It should be noted that in all the above papers the magnetic characterization was predominantly based on integral SQUID magnetometry.

In parallel to the experimental reports theoretical groups tried to shed light on the possible origin of the observed RT-ferromagnetism. An empirical coalescence model with magnetically polarized “spheres of influence" was inferred in the original work introducing the colossal magnetic moments [36]. The microscopic origin of such a magnetic polarization of the host was uncertain and later-on detailed synchrotron studies revealed no significant magnetic polarization of the Ga [105]. Theory was initially suggesting an antiferromagnetic order via sf-coupling which can be tuned to be ferromagnetic by electron doping [106]. More recently, Ga vacancies were held responsible for the ferromagnetism in Gd:GaN [107,108] and even in GaN, *i.e.*, without Gd doping [109]. This model was criticized because of the large number and high energy of formation of Ga vacancies and alternatively, interstitial N or O on octahedral sites were proposed to mediate the RT-ferromagnetism [110].

Summarizing, although frequently reported and less questioned, the existence of RT ferromagnetism has not yet been unambiguously established by experiments other than SQUID magnetometry. Similar to the Co:ZnO compound also for Gd:GaN an unambiguous interrelation of structural properties and magnetism is required to be substantiated experimentally.

## 3. Experimental Techniques

This review is focused on two wide-band-gap DMS materials, Co:ZnO and Gd:GaN, which were fabricated using a variety of deposition methods. Details of the preparation can be found in the respective sections. All samples presented here have undergone a comprehensive set of complementary experimental characterization techniques to yield integral as well as element-specific insight into their respective structural and magnetic properties. This approach aims at establishing an unambiguous correlation of the observed magnetic properties with the specialities of the respective structure. The detailed understanding is required to enable engineering these materials in such a way, that room-temperature spintronic devices become feasible. For that purpose a controlled and reproducible modification of the magnetic interaction in these materials is in order, e. g., by modifying the carrier concentration without changing the structure, e. g., by phase separation or dopant-clustering. In the following the experimental techniques which were employed to study the DMS samples are summarized.

### 3.1. Structural Properties

#### 3.1.1. Integral Methods

The standard structural characterization for all samples was done by means of X-ray diffraction (XRD). Monochromatic XRD was performed using a commercial high-resolution four-circle diffractometer. The device is equipped with a Göbel mirror to create a parallel X-ray beam and a Ge monochromator to provide monochromatic Cu $K_{\alpha 1}$ radiation (λ=1.540562 Å). Non-monochromatic XRD measurements were done with a Philips PANalytical X’Pert PRO using non-monochromatised Cu $K_{\alpha 1}$ and $K_{\alpha 2}$ radiation with a weighted average wavelength of 1,5418 Å together with an X’Celerator detector. The advantage of the latter diffractometer is the high intensity of the X-rays enabling a relatively sensitive probe for small nanocrystals within fairly short measuring times (∼2 h) compared to the monochromatic diffractometer where a comparable sensitivity to small nanocrystallites usually requires averaging times of a few days, see e. g, [48]. In all cases a logarithmic scale for the diffractogram is used to enable judging whether the sample is free of secondary phases in the form of small precipitates. Diffraction data on a linear scale which can be frequently found in the literature cannot constitute a proof of the absence of secondary phases. Even exercising greatest care, the detection limit of a small crystallite in a single crystalline matrix is a diameter of 2–4 nm. For structurally distorted or even amorphous nanophases the sensitivity of XRD is much worse. Therefore, XRD data can only serve to prove that a sample is free of secondary *crystalline* phases above 2 nm diameter at best, *i.e.*, after long averaging times or by synchrotron-based XRD like in [73].

#### 3.1.2. Element-specific Methods

An alternative approach to probe the structural properties can be based on X-ray absorption measurements (XAS). Using XAS offers the opportunity to study the material properties with element selectivity. By tuning to the respective characteristic absorption edges, the cationic and the anionic species as well as the dopant can be studied separately. The probably most widely used sub-technique to probe the structural properties is extended X-ray absorption fine structure spectroscopy (EXAFS) which probes the local pair-correlation function of the absorbing atom and thus the structural properties on a local scale averaged over all absorbing atoms [111]. However, the EXAFS amplitude can be reduced due to thermal or structural disorder as described by the dynamical and statical Debye-Waller factors entering the EXAFS analysis. While the local geometry such as next-neighbor distances can be directly extracted from the EXAFS, it is difficult to yield quantitative information about the percentage of the absorbing species being located on the ideal lattice site, since the contribution of eventual secondary phases formed by the absorbing species to the EXAFS cannot be assessed in a straightforward manner.

**Figure 1:**
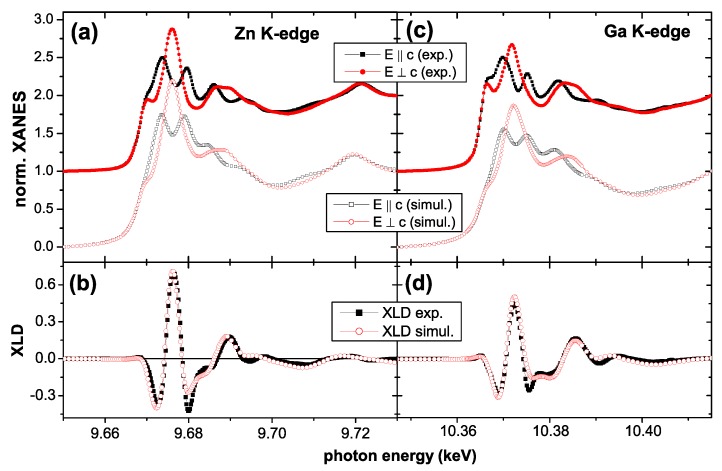
Experimental XANES spectra recorded with E∥c (black squares) or perpendicular E⊥c (red circles) at (a) the Zn *K*-edge of *c*-oriented ZnO and at (c) the Ga *K*-edge of *c*-oriented GaN (right). The experimental spectra are shown together with the respective simulations using the FDMNES code. The (b) and (d) show the respective difference between the two experimental (black squares) and simulated (red circles) spectra constituting the characteristic XLD of the ZnO and GaN wurtzite lattices.

Therefore, in the frame of this work another, less well-known XAS-based technique is used. Both materials in question, ZnO and GaN crystallize in the wurtzite structure, which has a uniaxial crystal symmetry with fairly directional bonding of strong covalent (GaN) or even ionic-like (ZnO) character. This leads to the fortunate situation that the crystal field of the anions splits the electronic state of the tetrahedrally coordinated cation and vice-versa so that there is a clear directional dependence of the density of unoccupied final states, which are probed by XAS, especially at the near-edge (XANES). All samples, which can be prepared as single crystalline or highly oriented material, can be measured with linear polarized X-rays in such a way, that the *E* vector of the light is either parallel (E∥c) or perpendicular (E⊥c) to the *c*-axis of the crystal. The difference between these two spectra, the so-called X-ray linear dichroism (XLD) has a characteristic shape for the wurtzite structure as shown in Figure 1 for the Zn *K*-edge of ZnO (a) and (c) and the Ga *K*-edge of GaN (b) and (d). In general, the structure of both compounds is quite alike, thus being indicative of the wurtzite structure; the slightly reduced size of the XLD for GaN indicates that the bonding is less ionic thus reducing the strength of the crystal field. Figure 1 also shows that these spectra together with the respective XLD can be simulated using the FDMNES code [112]. This approach has previously been chosen to study different DMS materials such as Mn:GaN [34], Gd:GaN [113], and Co:ZnO [49,62] and to quantitatively determine the amount of dopant atoms residing on cation lattice sites.

All XAS measurements presented here were taken at the ESRF beamline ID12 in total fluorescence yield [114]. It is critical to note, that in this work the hard X-ray regime was chosen to yield sensitivity over a *μ*m-depth scale, allowing full characterization of epitaxial films with a range of thicknesses as well as their interfaces to the substrate. In contrast, XAS the soft X-ray regime makes it particularly difficult to sense the interface or the entire volume of thick (more than ∼300 nm) epitaxial films, since total electron yield measurements probe only the first few nanometers of the film [115] and for the fluorescence yield a probing depth of approx. 100 nm can be achieved [116]. The XANES/XLD measurements were carried out at 300 K and a quarter wave plate [114] was used to flip the linear polarization of the synchrotron light from vertical (*i.e.*, E∥c) to horizontal (E⊥c); the angle of incidence was 10${}^{\circ }$ with respect to the sample surface. The XLD was taken as the direct difference of the normalized XAS with E⊥c and E∥c; the linear polarization was flipped forth and back three times at each energy point of the spectra. The XANES was derived from the weighted average of the two XAS spectra, *i.e.*, (2× XAS(E⊥c) + XAS(E∥c))/3.

### 3.2. Magnetic Properties

First, a few definitions regarding the magnetic properties shall be made which will be used throughout this review. The term *“ferromagnetic"* **(FM)** will only be used, if it can be experimentally shown by more than one experimental method, that long-range magnetic order with a remanent magnetization, magnetic anisotropy and a magnetic hysteresis are present. All these properties shall vanish at the Curie-temperature $T_{C}$ above which the system is paramagnetic exhibiting typical atomic magnetic moments of a few $\mu_{B}$ per atom.

The term *“paramagnetic"* **(PM)** will be used, if a non-interacting (except weak dipolar interactions) atomic moments of a few $\mu_{B}$ per atom are studied and both remanence and magnetic hysteresis are absent. Note, that also PM can be anisotropic, e.g., Co:ZnO [117,118].

The term *“superparamagnetic"* **(SPM)** will be used if a magnetic hysteresis and remanence are present at low temperatures which vanish above a certain temperature above which the paramagnetic response is indicative of large effective magnetic moments. This is typically asserted by the presence of a clear blocking behavior (separation of field cooled (FC) *versus* zero-field cooled (ZFC) M(T)-curves with a clear maximum in the ZFC curve) and an anhysteretic, S-shaped M(H)-curve even at elevated temperatures. SPM is typically present if a non-, or weakly (dipolar) interacting ensemble of small FM particles (ferromagnetic nanoparticles) is studied.

In the context of DMS materials it is crucial to note, that the vast majority of presented data throughout the literature is merely indicative of SPM rather than FM, with the exception of Mn:GaAs, where FM is well-established in the above sense. It should be also noted, that SPM in DMS systems can originate from different microscopic scenarios. On the one hand, if a coalescence model of bound magnetic polarons is considered as in [64], SPM-like signatures in integral magnetometry are likely. The “blocking temperature" would correspond to the temperature where the magnetic coupling between the magnetic polarons breaks down. A similar SPM-like magnetism is also inferred by a charge-transfer mechanism [119]. Within these scenarios, SPM would be an *intrinsic* property of the material. On the other hand, small ferromagnetic inclusions (“nanoparticles" or nanoclusters) more naturally constitute SPM behavior. Such nanoclusters can originate from different sources: (i) phase separation of dopant atoms, (ii) decoration of grain boundaries with dopant atoms beyond the solubility limit, (iii) magnetic contaminations due to sample-handling such as wafer cutting by the manufacturer, cleaving, improper tweezers-handling, improper sample mounting, or even using a marker-pen to label the samples on the backside, and (iv) dopant-rich regions in a dopant-poor host matrix such as in the Mn:Ge system, e.g., [120], often termed as “spinodal decomposition" [121,122]. Whereas (iv) can be considered as an *intrinsic* property of the material, (i) to (iii) will be regarded as *extrinsic* and therefore not as a real physical property of the material in question. Note, that it is experimentally extremely difficult to distinguish between SPM from a bound magnetic polaron model or (iv) and (i) to (iii), especially, if TEM experiments reveal no nanoclusters. Where phase separation was experimentally demonstrated, e.g., in [48,74], the successful search was tedious and facilitated by a combination of energy filtering TEM (EFTEM) and careful XRD.

#### 3.2.1. Integral Methods

Most of the exciting claims of unusual magnetic properties have the use of SQUID magnetometry in common, e.g., anisotropic ferromagnetism [54], colossal magnetic moments [36,98] or ferromagnetism in oxides like HfO${}_{2}$ [123]. The majority of the experiments for solid state samples is performed using the commercial SQUID magnetometer MPMS (XL) from Quantum Design. Using the MPMS SQUID is widely spread mainly due to its high degree of user-friendly automation and reliability as well as the lack of commercial alternatives. On the other hand, there are studies which highlight possible errors and artifacts in such magnetometric measurements like the influence of stainless-steel tweezers handling on the magnetic properties of HfO${}_{2}$ samples [124] or point out “possible pitfalls in search of magnetic order" for samples using sapphire substrates which can exhibit ferromagnetic signatures themselves [125]. The latter can be circumvented by individually checking the substrates prior to the deposition of the actual film like, e.g., in [47]. Other groups highlight possible contamination of the sample holder, typically clear drinking straws, and of other means of sample mounting while using a home-built SQUID [126]. Besides these issues, also inherent artifacts of the SQUID magnetometer are discussed stemming either from the second-order gradiometer to detect the magnetic flux [127] or the magnetic field control of the superconducting magnet [128].

All integral magnetometric results presented here were recorded with a SQUID magnetometer (MPMS XL5). Typically the measurements were carried out by applying the magnetic field in the plane of the sample (H⊥c). A typical measurement protocol contains first a M(H)-curve recorded from +4 T to -4 T and back at 300 K followed by a cool-down to 5 K under +4 T. Then another M(H)-curve is recorded at 5 K. Then the M(T)-dependence at 10 mT is measured while warming the sample from 5 K to 300 K (FC). At 300 K the sample is demagnetized and cooled down to 5 K in nominally 0 mT. Then another M(T)-curve is recorded at 10 mT (ZFC). This procedure assures, that an eventual magnetic hysteresis at 300 K is visible in the FC/ZFC curves by a clear separation of the two measurements. For all samples great care was taken to minimize the known artifacts of this machine [128]. In particular, the edges of the substrate were thoroughly cleaned to avoid ferromagnetic contamination. In addition, all angular dependent SQUID measurements (H⊥c *versus* H∥c, out-of-plane) were carried out on the same piece of sample inside the same SQUID sample holder (a clear drinking straw). The sample could be freely rotated inside the straw as described in [118].

In addition to the SQUID magnetometric measurements electron paramagnetic resonance (EPR) measurements were performed on a selection of samples. All EPR investigations have been carried out using a commercial Bruker X-band spectrometer. The maximum external field was 1.2 T. Typically, a cylindrical cavity was used and the resonance signal was recorded as a function of the polar angle Θ. For low temperature measurements a dynamic flow cryostat was used which enables measurements down to ∼5 K. Temperature and angular-dependent EPR measurements are adjuvant to unambiguously discriminate between ferromagnetism or (super-)paramagnetism.

#### 3.2.2. Element-specific Methods

The above integral magnetometries are inherently sensitive to the entire sample specimen. For EPR measurements possible paramagnetic impurities in any type of substrate contribute to the resonance spectrum, whereas for SQUID magnetometry the predominant contribution to the overall signal stems from the diamagnetic response of the substrate which is not detected by EPR. To detect a magnetic signal, which unambiguously stems from the DMS film and not from the substrate underneath, element specific techniques such as the X-ray magnetic circular dichroism (XMCD) are useful. XMCD offers the unique possibility to assign the magnetic response of a sample to a certain atomic species [91]. Typically DMS materials are studied using soft X-rays, *i.e.*, for Co:ZnO DMS the Co $L_{3/2}$-edges are studied, e. g., [48,62,63]. This has the inherent advantage of both substantial dichroic signal and quantitative analysis using the well-established XMCD sum-rules [129,130]. On the other hand, the probing depth of soft X-rays is limited as discussed above, so that for typical film thicknesses of a few hundreds of nanometer this technique may miss eventual secondary phases which may be present at the interface to the substrate. Therefore, throughout this work, hard X-rays of the beamline ID12 at the European Synchrotron Radiation Facility (ESRF) in Grenoble were used to record the XMCD signal in total fluorescence yield at the Co *K*-edge and the Gd $L_{3}$-edge, respectively.

**Figure 2:**
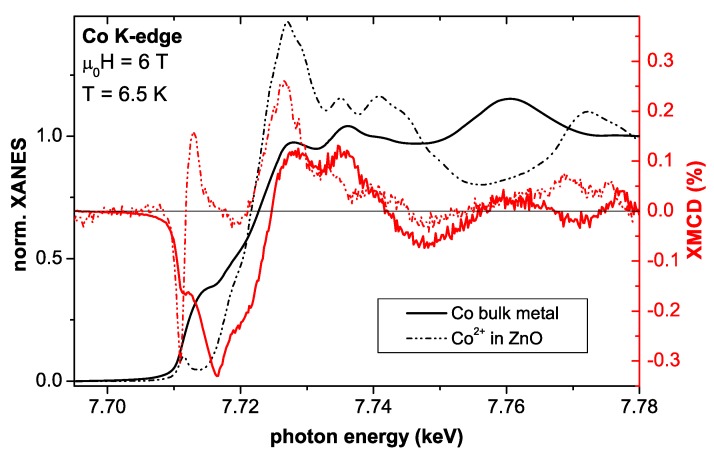
Experimental XANES (black) and corresponding XMCD (red) spectra recorded with left and right circular polarized light at the Co *K*-edge of bulk Co metal (full line) and *c*-oriented Co:ZnO (dash-dotted line) at 6.5 K and 6 T. The fine structure of the XANES and the XMCD is significantly different for the two Co species.

The XMCD measurements were taken at 6.5 K as the direct difference of XANES spectra recorded with right and left circular polarized light for H=6 T typically under grazing incidence of $15^{\circ }$. To minimize artifacts, the direction of the external magnetic field was reversed as well. Typical XANES and XMCD spectra at the Co *K*-edge for metallic bulk Co and a Co:ZnO film from [49] are exemplarily shown in Figure 2. The size of the XMCD at the Co *K*-edge is very small (about 0.3% of the edge jump) for both Co species. It is further only sensitive to the orbital fraction of the magnetic polarization of the 4*p*-like states. Nonetheless, it can provide valuable information about the magnetic state of the dopant atomic species throughout the entire film thickness. Descrepancies in the fine structure of both XANES and XMCD for the two Co species in Figure 2 are significant and characteristic energies can be found, where either metallic Co or Co:ZnO can be probed. Element specific M(H)-curves were typically recorded at photon energies of such characteristic features in the XMCD spectra (e. g. the XMCD at the pre-edge feature of Co:ZnO or the maximum XMCD of Co metal, where Co:ZnO shows no XMCD) by recording the difference of the X-ray absorption between left and right circular polarized light as a function of the external field. For each field value the circular polarization was switched forth and back to minimize effects which may originate from eventual drifts.

It should be noted, that according to the best knowledge of the author, there are few if any other beamlines operational beyond ID12, where it is possible to record the *K*-edge XMCD of a dilute system with reasonable accuracy, whereas XMCD at the Co *K*-edge of Co metal was also measured at other synchrotrons, e.g., [131,132,133]; where the spectral features are identical to the ones in Figure 2, but only the XMCD published in [133] yields a comparable quality. Although this limits the availability to the general community, the findings summarized here shall demonstrate that valuable information can be extracted from *K*-edge XMCD spectra, since they are more sensitive to the local structural arrangement than the $L_{3/2}$-edges. This is due to the fact that the final states at the $L_{3/2}$-edges, *i.e.*, the 3d bands are narrow near the Fermi level, so that they do not carry much information from the surrounding atoms. In contrast, at the *K*-edge the broader *p* conduction band is probed.

## 4. Co-doped ZnO Epitaxial Films

A comprehensive set of Co-doped ZnO samples was available for the present work comprising samples fabricated by three different techniques in four different institutions. Pulsed laser deposition (PLD) was either carried out at finite oxygen partial pressure (oPLD) in off-axis geometry at the Pacific Northwest National Laboratory (PNNL) or in an inert gas atmosphere (iPLD) in on-axis geometry at the Walther-Meißner Institut (WMI). Further results from the PNNL samples can be found in References [47,49,75,76] and some of the WMI samples are discussed in Reference [48]. Reactive magnetron sputtering (RMS) from metallic Zn/Co targets was used for growth under various oxygen partial pressures at the Universität Duisburg-Essen (UDE). In addition, a high-quality ZnO film was grown on a Mg:ZnO buffer layer by molecular beam epitaxy (MBE) on c-plane sapphire at the Walter-Schottky Institut (WSI) which was used for Co-ion-implantation at the Forschungszentrum Dresden-Rossendorf (FZR). This set of samples was already used in [72] to establish quality indicators for Co:ZnO by means of XANES, XLD and XMCD.

### 4.1. Basic Structural Properties

**Figure 3:**
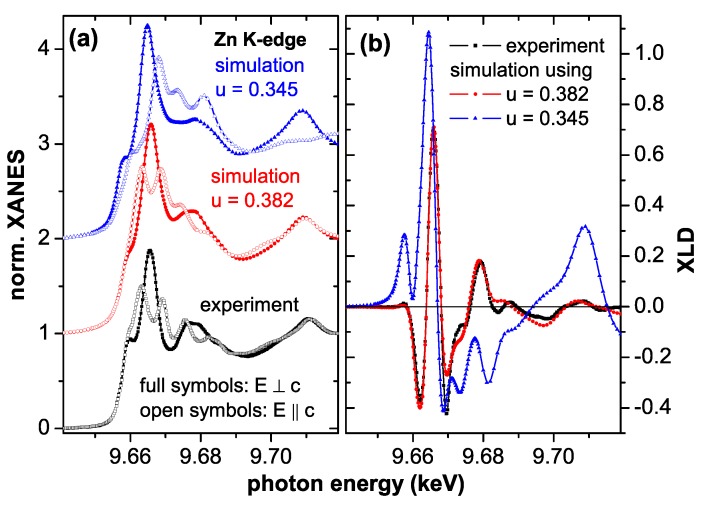
(a) XANES spectra recorded at the Zn *K*-edge with E∥c (open symbols) and E⊥c (full symbols) and corresponding XLD (b). The experimental data (black) were recorded at 300 K under 10${}^{\circ }$ grazing incidence. The simulations using the FDMNES code were done using the bulk lattice parameters and two different *u*-parameters, u=0.382 (red) and u=0.345 (blue).

**Figure 4:**
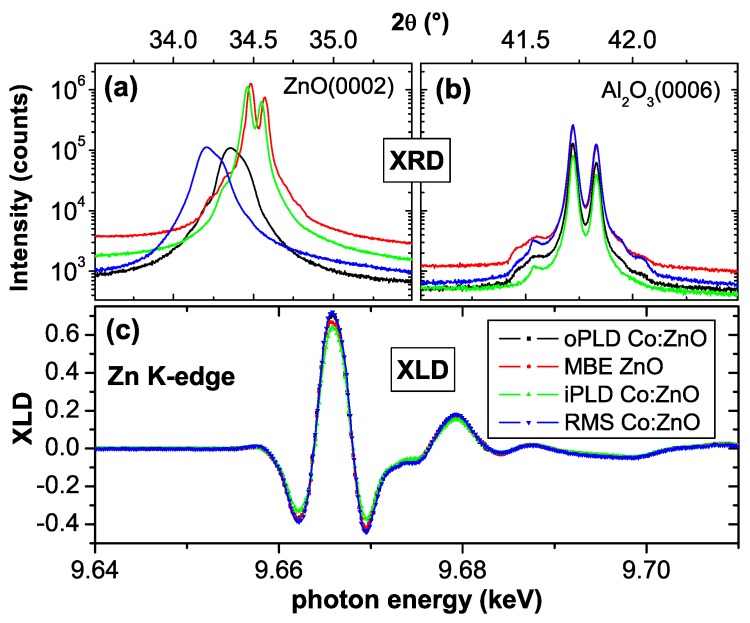
XRD *ω*-2*θ*-scans for (a) the ZnO(0002) and (b) the Al${}_{2}$O${}_{3}$(0006) reflection of three different Co:ZnO samples together with a MBE ZnO reference using non-monochromatized X-rays. (c) displays the respective XLD recorded at the Zn *K*-edge.

ZnO crystallizes in the wurtzite structure, which can be composed of a hcp cation and anion sublattice, respectively, which are shifted with respect to each other by the dimensionless *u*-parameter along the *c*-axis. For ideal tetrahedral coordination the *u*-parameter is 0.375 (=3/8). The bulk lattice constants are a=3.2459 Å and c=5.2069 Å, and a *u*-parameter of 0.382 is reported [134]. However, the initial theoretical prediction of RT ferromagnetism in Co:ZnO used a *u*-parameter of 0.345 [29], a value which was also used in more recent work [135] citing earlier work [136], where, however, u=0.382 is reported. To avoid any confusion regarding the correct *u*-parameter, the XLD at the Zn *K*-edge was measured for virtually all studied films and a representative spectrum is shown in Figure 3 (a). Together with simulations using the FDMNES code [112] this can be used to unambiguously determine the *u*-parameter. It is obvious from Figure 3 that a *u*-parameter of 0.382 fits the experimental data very well, whereas u=0.345 leads to significant deviations, especially in the XLD in Figure 3 (b). We therefore find for all Co:ZnO films in question u=0.382 in agreement with the findings in Reference [134,136]. It should be noted that the high-quality MBE ZnO film from the WSI exhibits identical XANES and XLD spectra at the Zn *K*-edge before Co-ion implantation as the Co:ZnO films, see Figure 4.

Besides studying the XLD at the Zn *K*-edge, all films were routinely measured using XRD. Figure 4 summarizes *ω*-2*θ*-scans of the ZnO(0002) reflection (a) in comparison to the Al${}_{2}$O${}_{3}$(0006) reflection of the substrate. The samples in question are a high quality ZnO reference film grown by molecular beam epitaxy (MBE) as well as three different Co-doped ZnO films. Two samples were grown by pulsed laser deposition (PLD), one under Ar partial pressure (iPLD) and one in an Ar/O mixture (oPLD). One Co:ZnO film was prepared using reactive magnetron sputtering (RMS). The RMS and the oPLD sample contain 10% of Co and are about 100 nm thick, the iPLD sample contains only 5% of Co and is 350 nm thick. Details of the sample preparation can be found in Reference [72]. From Figure 4 it can be seen, that all sapphire substrate reflections fall on top of each other, whereas the three Co-containing ZnO films exhibit a shifted ZnO reflection to lower angles, *i.e.* larger *c* lattice parameters increasing with Co content. Further, the two thinner films exhibit a slightly broader ZnO reflection. Note, that the presence of Cu $K_{\alpha 1}$ and $K_{\alpha 2}$ radiation leads to a double-peak structure, which cannot be resolved from a certain full-width at half maximum (FWHM) pretending a broader reflection. In Figure 4 (a) the oPLD and the RMS sample should have an intrinsic FWHM of about half of the visible reflection. No other reflection indicative of a secondary phase was observed. Figure 4 (c) displays the respective XLD signals of all four samples recorded at the Zn *K*-edge. The self-absorption due to the film thickness has been corrected. All four samples exhibit about the same size of the XLD signal indicating that virtually all Zn cations are located on ideal lattice sites for wurtzite ZnO. The increase in the *c* lattice parameter is not visible in the XLD, which is consistent with FDMNES simulations, indicating a weak dependence of the XLD on lattice distortions of the order of 1%, especially if the distortion is volume-conserving (not shown). The XLD of the oPLD sample has already been used in Reference [49] and in Figure 3 to adjust the FDMNES simulations to the experimental spectra.

Therefore, it can be concluded that four different preparation methods can result in (Co:)ZnO films which exhibit virtually ideal bulk-like ZnO lattice constants, a *u*-parameter of 0.382, and a small increase along the *c*-axis which depends on the film thickness and Co concentration. An XLD signal at the Zn *K*-edge of -0.4 to +0.7 is therefore indicative of an excellent structural quality on the local scale. In combination with a small FWHM also the long-range crystallographic order can be assessed to be excellent (MBE and iPLD) to very good (oPLD and RMS). It is worth to note, that RMS can lead to a comparable high structural quality as PLD for the growth of ZnO epitaxial films under optimized growth conditions.

### 4.2. Paramagnetic Co:ZnO Films

In the following the structural and magnetic properties of PM Co:ZnO shall be summarized. Exemplarily, the three Co:ZnO samples shown in Figure 4 will be discussed.

#### 4.2.1. Typical XANES/XLD Signatures

The XANES recorded at the Co *K*-edge of Co:ZnO has recently been studied to assess the valency of the Co and its local structural environment [49,62,67,72,75,76,118]. The *K*-edge is particularly sensitive to the local structure and valency since *p* final states are probed which have a larger spatial extent compared to the *d* states probed at the $L_{3/2}$-edges as done, e. g., in [48,62,63]. Reference XANES allow to distinguish between metallic (elemental) Co(0) (Figure 2), Co in Co:ZnO [49,72], and Co in either CoO or Co${}_{3}$O${}_{4}$ [67].

**Figure 5:**
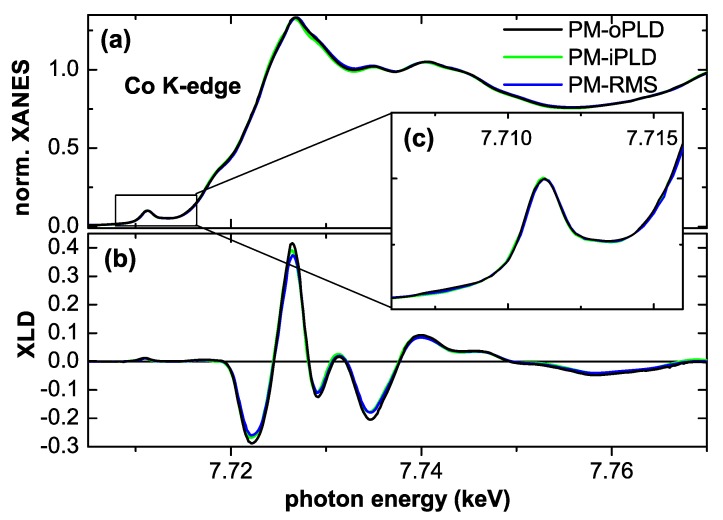
(a) normalized XANES at the Co *K*-edge of three different PM Co:ZnO samples and (b) the respective XLD signal. (c) enlarges the pre-edge feature of the XANES (see text).

Figure 5 (a) displays the normalized XANES of three PM Co:ZnO samples (RMS, iPLD, and oPLD) already shown in Figure 4. All three samples exhibit virtually identical XANES and the respective XLD is of the same size as shown in (b). This indicates that all samples have a comparably high degree of substitutional Co incorporation on Zn lattice sites as corroborated by simulations of the XLD of the oPLD sample in [49]. Figure 5 (c) enlarges the pre-edge feature associated with a 1s→(3d,4p) transition, which is separated by a local minimum from the main peak, which is predominantly a 1s→4p transition. The depth of this valley can be taken as a measure of the valency of the Co [72]. It is obvious from Figure 5 that all three samples have a virtually ideal incorporation of Co${}^{2+}$ on Zn lattice sites and thus the intrinsic properties of Co:ZnO can be probed on these specimens since they are devoid of secondary phases or significant amounts of interstitial or elemental Co.

#### 4.2.2. SQUID Results

By now we have established the phase pureness of three Co:ZnO epitaxial films fabricated by three different deposition techniques in three different labs. They were studied using XRD, XANES and XLD and their structural quality has been demonstrated on the global and the element-specific local scale. Integral magnetometry on these specimens therefore shall reveal their intrinsic magnetic properties. Note, that any contamination of such samples would only mimic FM-like behavior but is not able to mask FM in Co:ZnO.

**Figure 6:**
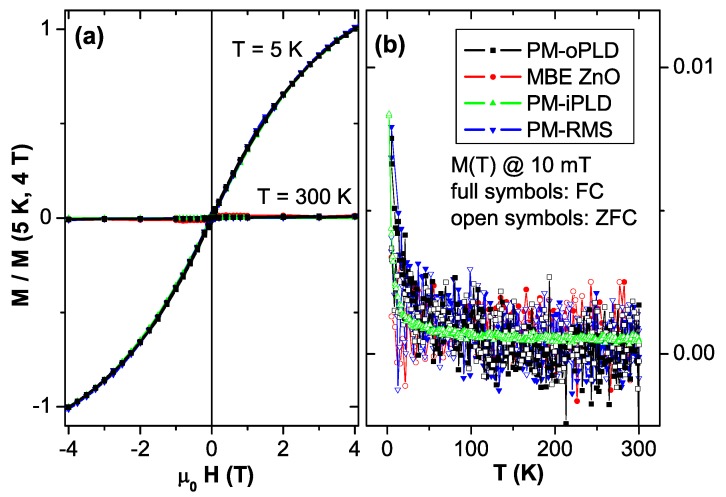
SQUID measurements on three Co:ZnO samples and the MBE ZnO reference. (a) displays M(H)-curves at 300 K and 5 K and (b) the respective M(T) in FC and ZFC conditions. All data are normalized to M(5 K, 4 T) and the diamagnetic background of the substrate has been derived from the high-field behavior of the 300 K data and subtracted from all data sets.

Figure 6 illustrates the integral magnetic properties applying the magnetic field in the film plane (H⊥c) of the three Co:ZnO samples *a posteriori* justifying to term them PM. M(H)-curves have been measured at 300 K and at 5 K (a) and the respective M(T) measurements under FC and ZFC conditions (b). The diamagnetic background from the substrate has been derived for each sample by the slope of the M(H)-curve at 300 K at large magnetic fields and has been subtracted from all data sets. For ease of comparison, the displayed magnetization is further normalized by the magnetization measured at 5 K and 4 T for each sample. The three samples exhibit identical M(H) behavior (a), devoid of any opening in the M(H)-curves at 5 K or any sizable magnetization at 300 K in excess of the detection limit of the SQUID instrument [128]. The M(T)-curves of the three PM samples strongly overlap (b), revealing no separation between the field-cooled (FC) and zero-field-cooled (ZFC) data. It should be noted that the MBE ZnO sample exhibits identical M(H) behavior at 300 K and 5 K, which is hardly visible in Figure 6 (a). This is indicative of a very low degree of contamination of both ZnO and sapphire with paramagnetic impurities which is corroborated by the absence of the up-turn of the M(T) data at low temperatures in Figure 6 (b). Adding 5% to 10% of Co to ZnO without inducing phase separation obviously induces only paramagnetism as evidenced by SQUID.

#### 4.2.3. XMCD Results

At first sight it may appear surprising that such high concentrations of Co can be ideally incorporated in ZnO without inducing more than PM. Since it is the aim of material scientists to induce FM order, the evidence of PM of the intrinsic Co:ZnO system needs a closer inspection by complementary techniques. First, the source of the PM response in the SQUID shall be verified by means of element-specific magnetometry. XMCD measurements at the Co *K*-edge of Co:ZnO have already been reported [49,67,72,137] and characteristic spectral features can serve to distinguish between the magnetic response of elemental Co and of Co${}^{2+}$ in ZnO as illustrated by Figure 2.

**Figure 7:**
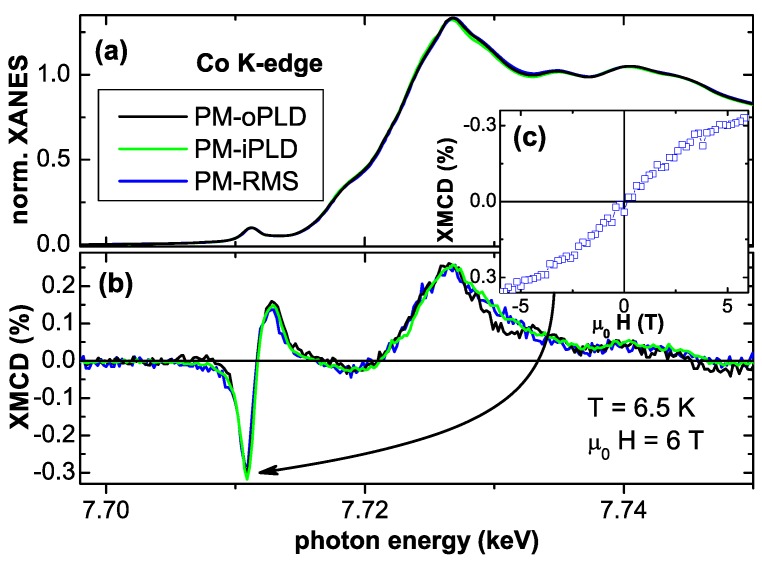
(a) normalized XANES and respective XMCD (b) at the Co *K*-edge of three PM Co:ZnO samples recorded at 6.5 K applying ±6 T in the film plane. (c) shows the element specific M(H)-curve as measured at the photon energy of the pre-edge feature of the PM-RMS sample.

Figure 7 displays the normalized XANES (a) and respective XMCD (b) recorded at 6.5 K and applying ±6 T in the film plane (H⊥c). The inset (c) shows the size of the dichroic signal recorded at the pre-edge feature of the PM-RMS sample as a function of the external field (element specific M(H)-curve). The functional dependence is identical to the element specific M(H)-curve recorded at the main peak, but with opposite sign (not shown). All three samples exhibit a maximum XMCD signal of 0.3% at the pre-edge in agreement with [67] which can be taken as indicative of the intrinsic PM response of the Co sublattice [72]. A reduced size of the XMCD signal for a comparable Co concentration is in turn indicative of non-ideal incorporation of Co${}^{2+}$ [72,137]. Note, that in [137] the XMCD is displayed with opposite sign. Although the element specific magnetometry by means of XMCD corroborates the PM of the Co:ZnO samples, see Figure 7 (c), a quantitative determination of the size of the magnetic moment is not possible due to the lack of suitable sum rules. In addition the maximum XMCD signal also depends on the Co concentration [118].

#### 4.2.4. EPR Results

So far, integral as well as element specific magnetometry has only revealed PM as the intrinsic property of Co:ZnO in the concentration range of 5% to 10% of Co. This compares well with the fact that Co${}^{2+}$ is a well-known 3$d^{7}$ PM impurity in ZnO. Early investigations were restricted to EPR studies [138] or optical absorption measurements [139] in which Co${}^{2+}$ was present at the impurity level (10 ppm Co in Reference [139]) in ZnO single crystals.

**Figure 8:**
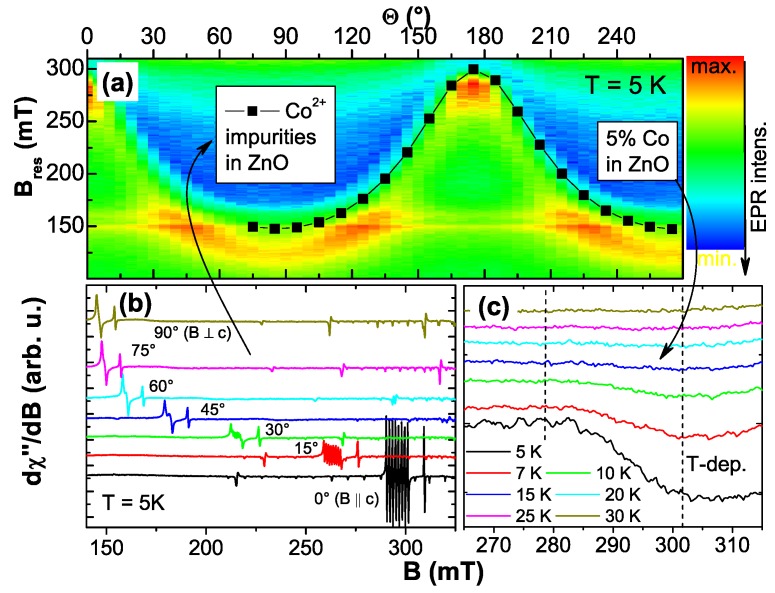
(a) EPR color-code plot recorded at 5 K of a 5% Co-doped ZnO sample grown by RMS as a function of the polar angle Θ in comparison with Co${}^{2+}$-impurities in bulk ZnO (black squares). The hyperfine-split octet of the Co${}^{2+}$ impurities is shown in (b); the broad resonance feature of the 5% Co:ZnO film vanishes quickly with temperature (c).

Figure 8 compares two EPR measurements recorded at 5 K as a function of the polar angle Θ for two different types of samples. The color-coded diagram (a) represents the findings for a ∼1 *μ*m thick 5% Co:ZnO film grown by RMS. A broad (∼100 mT) resonance with uniaxial behavior is visible, which quickly vanishes upon increasing temperature as seen in Figure 8 (c). Note that the same resonance could be detected for thinner Co:ZnO samples with 5% and 10% Co. However, due to the reduced number of Co atoms and the increased broadening at 10%, the signal was much less pronounced. For comparison, a commercially available ZnO *c*-plane substrate (CrysTec GmbH) was measured under identical conditions. This substrate contains various types of paramagnetic impurities, amongst them Co${}^{2+}$, confirmed by the presence of an hyperfine-split octet with uniaxial anisotropy, see Figure 8 (b). This anisotropic hyperfine splitting serves to unambiguously identify Co${}^{2+}$ (nuclear spin I=7/2), see Reference [140]. The center of gravity of this octet is shown as black squares in Figure 8 (a). The good agreement between the two experiments indicates that the *g*-factors ($g_{∥}$ and $g_{⊥,\mathrm{eff}}$) used to model the anisotropic paramagnetic behavior according to [117,138] do not significantly change as a function of Co concentration, independent of whether the Co is present at the impurity level or at concentrations as high as 5%. On the other hand, the hyperfine-splitting is not visible for the 5% sample and the resonance line is strongly broadened due to (weak) dipolar interactions. Such a broadening has already been reported [141]. However, we do not find any evidence for exchange pairs as in [141]. These findings highlight the fact, that no other interactions beyond weak dipolar coupling exists in phase-pure Co:ZnO samples.

#### 4.2.5. Anisotropic Paramagnetism

Having demonstrated PM in 5% to 10% Co:ZnO it should be feasible to model the magnetic properties with the well-known effective S=3/2 spin Hamiltonian for Co${}^{2+}$ (3$d^{7}$) impurities in ZnO [117,138,139]:

(1) $${\hat{H}}_{spin}=\mu_{B}g_{∥}H_{z}S_{z}+\mu_{B}g_{⊥}\left(H_{x}S_{x}+H_{y}S_{y}\right)+DS_{z}^{2}$$

where two *g*-factors $g_{∥}=2.238$ (H∥c) and $g_{⊥}=2.276$ (H⊥c) and the zero-field splitting constant *D* stemming from the spin-orbit (SO) interaction capture the magnetic state. The respective ${}^{4}A_{2}$ ground state alone is responsible for the magnetic response in SQUID and EPR experiments. It is SO-split by 2D=0.684 meV forming two levels, $E_{1/2}$ and $E_{3/2}$ as measured by EPR [138] and optical measurements [139] on Co impurities in ZnO single crystals and more recently confirmed for epitaxial 0.28% Co:ZnO films [117].

**Figure 9:**
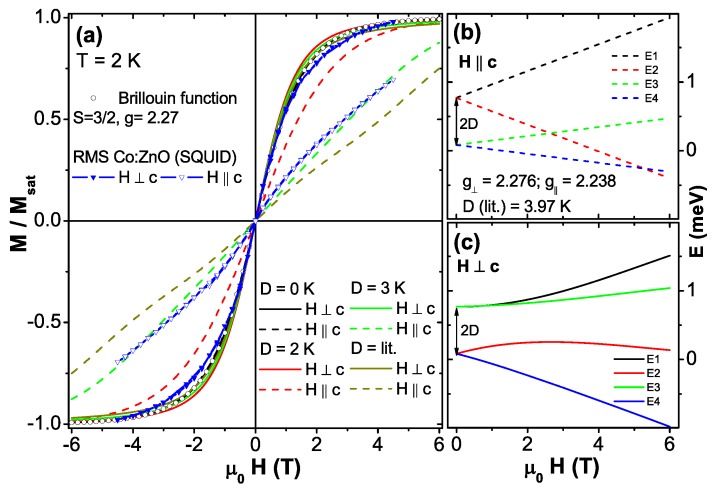
(a) Calculated M(H)-curves at 2 K for H∥c (dashed lines) and H⊥c (solid lines) using the effective spin model and different values of the zero field splitting *D*. The Brillouin function is given for comparison (open circles) as well as experimental SQUID data of the PM-RMS sample (blue triangles). The M(H)-curves were derived from the calculated energy levels for H∥c (b) and H⊥c (c) using literature values.

Equation 1 serves to calculate the M(H)-curves in Figure 9 (a) by thermally occupying the energy levels of the S=3/2 manifold $|M_{s}\rangle =|-3/2\rangle$ ...|3/2〉 by the matrix $\langle M_{s}|{\hat{H}}_{spin}|M_{s}\rangle$ for H∥c ($H=H_{z}$, Figure 9 (b)) and H⊥c ($H=H_{x}$, Figure 9 (c)), respectively. For H∥c the matrix is diagonal and the energy levels are given by:

(2) $$\;E_{1/2}=\frac{9D}{4}\pm \frac{3}{2}\mu_{B}g_{∥}H_{z}\;E_{3/4}=\frac{D}{4}\pm \frac{1}{2}\mu_{B}g_{∥}H_{z}$$

The four energy levels given by Equation 2 are plotted in Figure 9 (b) using the literature values for $g_{∥}=2.238$, $g_{⊥}=2.276$, and D=0.342 meV (=3.97 K). These parameters were recently derived by modern crystal field theory [142]. At moderate magnetic fields the lowest energy level $E_{4}$ is S=1/2-like. At high magnetic fields the S=3/2-like $E_{2}$-level becomes lower in energy. For H⊥c the analytical diagonalization of the matrix yields:

(3) $$E_{1}=\frac{1}{2}\mu_{B}g_{⊥}H_{x}+\frac{5}{4}D+\sqrt{\mu_{B}^{2}g_{⊥}^{2}H_{x}^{2}-Dg_{⊥}\mu_{B}H_{x}+D^{2}}$$

$$E_{2}=\frac{1}{2}\mu_{B}g_{⊥}H_{x}+\frac{5}{4}D-\sqrt{\mu_{B}^{2}g_{⊥}^{2}H_{x}^{2}-Dg_{⊥}\mu_{B}H_{x}+D^{2}}$$

$$E_{3}=-\frac{1}{2}\mu_{B}g_{⊥}H_{x}+\frac{5}{4}D+\sqrt{\mu_{B}^{2}g_{⊥}^{2}H_{x}^{2}+Dg_{⊥}\mu_{B}H_{x}+D^{2}}$$

$$E_{4}=-\frac{1}{2}\mu_{B}g_{⊥}H_{x}+\frac{5}{4}D-\sqrt{\mu_{B}^{2}g_{⊥}^{2}H_{x}^{2}+Dg_{⊥}\mu_{B}H_{x}+D^{2}}$$

The energy levels resulting from Equation 3 are plotted in Figure 9 (c). In this case the lowest energy level $E_{4}$ is S=3/2–like and no crossing of the energy levels occurs. In the following the discussion is limited to the role of the zero-field splitting *D*. Figure 9 (a) shows the dependence of the anisotropy of the M(H)-curves on the strength of the zero-field splitting *D* calculated for T=2 K. For that purpose the magnetization $M=-\;{\left(\partial F/\partial H\right)}_{T}$ of the magnetic free energy $F=-\;k_{B}T\ln Z$ using the partition function $Z=\sum_{i}e^{-E_{i}/k_{B}T}$ was calculated using the energy levels shown in Figure 9 (b) and (c) for different values of *D* ranging from 0 K to the literature value of 3.97 K for H⊥c (solid lines) and H∥c (dashed lines). For comparison, the Brillouin function $B_{S}$ for S=3/2 and $g_{⊥}=2.276$ is shown as open black circles. As expected, the anisotropy decreases for decreasing *D*. In the limit of D=0 K the M(H)-curves calculated from the Brillouin function and the effective spin model are virtually identical. On the other hand, the shape of the M(H⊥c)-curve does hardly change with *D*-only a slightly increased curvature is visible and all M(H⊥c)-curves are rather similar to the Brillouin function explaining why experimental data could be rather successfully modeled that way, e. g., in [49,62].

The M(H)-curves for H∥c show a decreasing slope with increasing *D*, thereby being predominantly responsible for the increase in anisotropy. Figure 9 (a) also includes experimental SQUID data of the PM-RMS sample as blue triangles which is representative for all studied PM 10% Co:ZnO samples [118]. It is obvious that the SQUID data can be modeled well by D∼3 K, *i.e.*, *D* is reduced to 75% of the well-established literature value. A detailed discussion can be found in [118].

#### 4.2.6. Antiferromagnetic Co-O-Co Interaction

**Figure 10:**
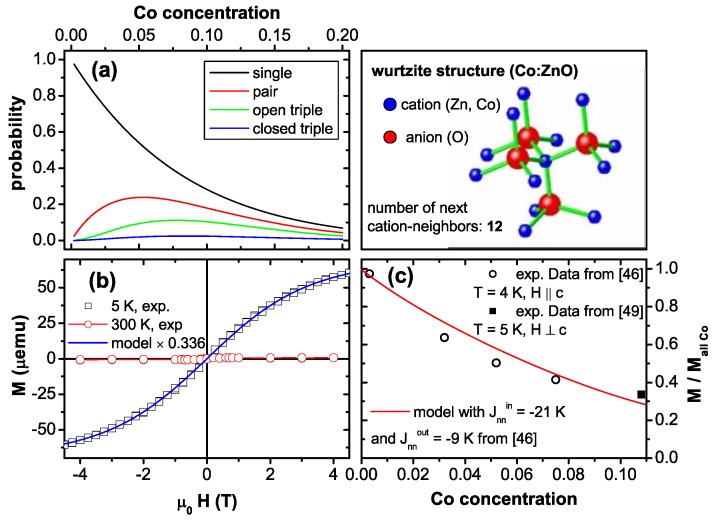
(a) Abundancies for various Co-O-...-configurations according to [143] for the cationic sublattice of wurtzite ZnO having 12 next cation neighbors. (b) Quantitative modeling of the SQUID data of 10.8% Co:ZnO (PM-oPLD) at 5 K with H⊥c indicating only 33.6% of the expected magnetization. (c) Reduction of M with respect to the expected value as a function of Co concentration inferring antiferromagnetic Co-O-Co interactions.

So far, it has been demonstrated that PM Co:ZnO films seem to follow the expectations known from Co impurities in ZnO and only dipolar interactions are evidenced by EPR. However, while the shape and anisotropy of the M(H)-curves could be nicely modeled, quantitative deviations in the size of the magnetization have been found [46,49]. To quantitatively analyze the magnetization, a statistical distribution of the Co atoms on Zn lattice sites will be assumed, since XLD has demonstrated that Co occupies almost exclusively such lattice sites, see Figure 5 and [49]. The wurtzite lattice of ZnO consists of two hcp sublattices for cations and anions, respectively, so that the statistics for the cationic sublattice is identical to the equations derived by Behringer for the hcp lattice [143]. Figure 10 (a) displays the abundance (probability) of isolated Co (singles), Co-O-Co (pairs) and two possible triples as a function of Co concentration for the cationic sublattice of wurtzite ZnO with 12 next cation neighbors as sketched in Figure 10. It is obvious, that the abundance of Co singles quickly goes down with increasing Co concentration, e. g., it is reduced to ∼28% for 10% Co:ZnO.

Figure 10 (b) displays experimental SQUID data measured at 5 K (black squares) and at 300 K (red circles) of the PM-oPLD sample together with a quantitative modeling according to Figure 9 (a). For this sample the number of Co atoms has been experimentally determined [49] so that the modeling can be quantitative; different from [49], where the Brillouin function with an effective magnetic moment of 4 $\mu_{B}$/Co was used, the effective spin model with an effective magnetic moment of 3.14 $\mu_{B}$/Co has been taken. The expected magnetization has to be reduced by a factor of 0.336 to fit the data. This “lack of magnetization" is compared to experimental data and respective modeling of antiferromagnetic Co-O-Co interactions found in [46] in Figure 10 (c) indicating that over the entire concentration range from 0.28% to 10.8% of Co doping, the drop in measured magnetization can be fitted well by assuming antiferromagnetic coupling of Co-O-Co pairs and negligible contributions from larger Co-O-...-configurations. Whereas the antiparallel spin-alignment of pairs can be directly inferred from the experimental data shown in Figure 10, it is more difficult to assign the PM response to the singles only. The abundance of ∼28% for singles at 10% of Co is slightly smaller than the reduction of the magnetization of 33.6% as found in Figure 10 (b). On the other hand, it is known that these epitaxial films grow in a nanocolumnar nanostructure so that the probability for singles is increased to about 31% to 36%, depending on the diameter of the nanocolumns as shown by Monte-Carlo simulations [144]. It is therefore hard to decide, whether the larger Co-O-...-configurations contribute to the integral magnetic response as discussed in [46] or not. At more elevated temperature there are further deviations from the expectations of the effective spin model, especially at higher Co concentrations [118], which can be attributed to these larger Co-O-...-configurations.

In summary, we have demonstrated that structurally well-defined Co:ZnO epitaxial films behave PM. This has been accomplished by complementary structural and magnetic techniques using lab-based as well as synchrotron methods. Isolated Co dopant atoms exhibit the typical single-ion anisotropy known from Co${}^{2+}$ impurities in ZnO, however, the zero-field splitting *D* is reduced by 75% to 3 K. Quantitative magnetometry reveals antiferromagnetic interactions of Co-O-Co pairs. The contribution of larger Co-O-...-configurations to magnetometric data is minor. The only unambiguous sign on long range interactions is the broadening of the EPR line-width, which is however only indicative of weak dipolar coupling. Therefore, no intrinsic FM interactions were evidenced.

### 4.3. Superparamagnetic Co:ZnO Films

Having established that intrinsic Co:ZnO, *i.e.*, phase-pure, virtually defect-free, and structurally well-defined Co:ZnO is PM, the question remains, how to induce FM or at least SPM properties in this DMS material. Since here the emphasis is put on synchrotron-based methods, crystallographically not well-oriented Co:ZnO samples will be excluded, *i.e.*, nanoparticles or nanorods of Co:ZnO. However, it should be noted, that the spectral shape of the XMCD at the Co *K*-edge as reported in [67] can serve as quality indicator for the phase-pureness of Co:ZnO nanoparticles or -powders. In the following three different approaches to alter the magnetic properties of epitaxial Co:ZnO films will be discussed. It should be stressed that for all three preparation techniques, which are discussed in the following, it was possible to fabricate both intrinsic PM (as shown in Section 4.2) and phase-separated SPM samples. The differences in preparation between PM and SPM samples were minor: for RMS samples the oxygen content in the sputter gas was reduced which alters the carrier concentration [145]; for the iPLD samples the substrate was changed from sapphire to ZnO intended to improve the crystallinity; or for the oPLD samples the orientation of the sapphire substrate was changed from *c*- to *r*-plane sapphire. As a fourth SPM sample, the MBE ZnO epitaxial film was implanted with Co${}^{+}$ ions under conditions known to lead to phase separation of small Co nanoclusters evidenced by synchrotron-based XRD [73].

**Figure 11:**
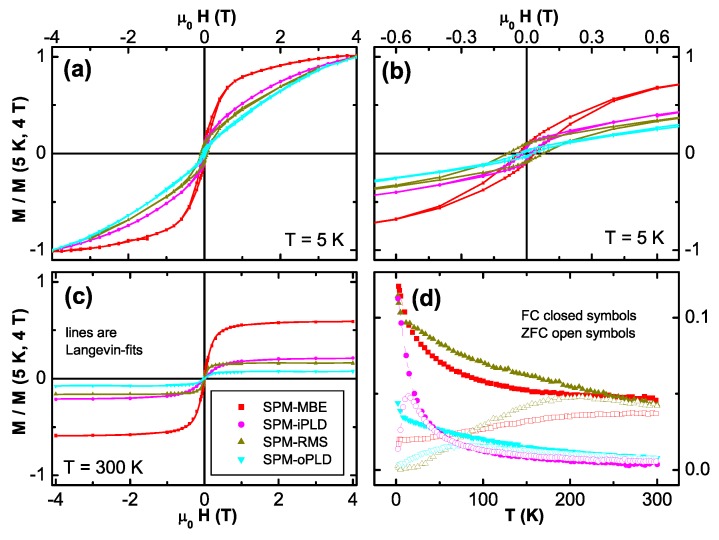
SQUID measurements on four SPM Co:ZnO samples. (a) displays the M(H)-curves at 5 K and the low field region is enlarged in (b) exhibiting a clear hysteretic behavior. (c) shows the anhysterestic, s-shaped M(H)-curves at 300 K and (d) the respective M(T) in FC and ZFC conditions. All data are normalized to M(5 K, 4 T) and the diamagnetic background of the substrate has been derived from the high-field behavior of the 300 K data and subtracted from all data sets.

Figure 11 summarizes the integral magnetic properties measured by SQUID applying the magnetic field in the film plane of the four SPM Co:ZnO samples. The diamagnetic background from the substrate has been derived for each sample by the slope of the M(H)-curve at 300 K at large magnetic fields and has been subtracted from all data sets. For ease of comparison, the displayed magnetization is further normalized by the magnetization measured at 5 K and 4 T for each sample. M(H)-curves have been measured at 5 K (a) exhibiting hysteretic behavior, as the magnification of the low-field region reveals (b). The respective M(H)-curves recorded at 300 K (symbols in Figure 11 (c)) show a pronounced S-shape but are anhysteretic. They can be fitted well using a Langevin function (lines in Figure 11 (c)). Such a fit yields an average supermoment and an average particle diameter. We derive 5100 $\mu_{B}$ (∼4 nm) for the SPM-MBE, 2500 $\mu_{B}$ (∼3 nm) for the SPM-iPLD, 10000 $\mu_{B}$ (∼5 nm) for the SPM-RMS, and 5000 $\mu_{B}$ (∼4 nm) for the SPM-oPLD sample, where the particle diameters given in parentheses are calculated assuming the magnetic species is metallic hcp Co (1.7 $\mu_{B}$/atom). The presence of SPM is further evidenced in Figure 11 (d), which summarizes the temperature-dependent magnetization data. The M(T) dependencies show a clear separation between the FC and the ZFC curves and varying *T* values for the maxima in the ZFC-M(T) behavior which can be taken as indication for different blocking temperatures $T_{B}$ consistent with the respective particle diameters inferred by simple Langevin-fitting. It should be noted, that $T_{B}$ also depends on the effective anisotropy of the clusters, therefore, no one-to-one correlation between supermoment and $T_{B}$ can be expected. Having established SPM behavior by integral SQUID magnetometry, the question remains to be addressed whether in the sense of Section 3.2 the SPM is of intrinsic or extrinsic origin.

**Figure 12:**
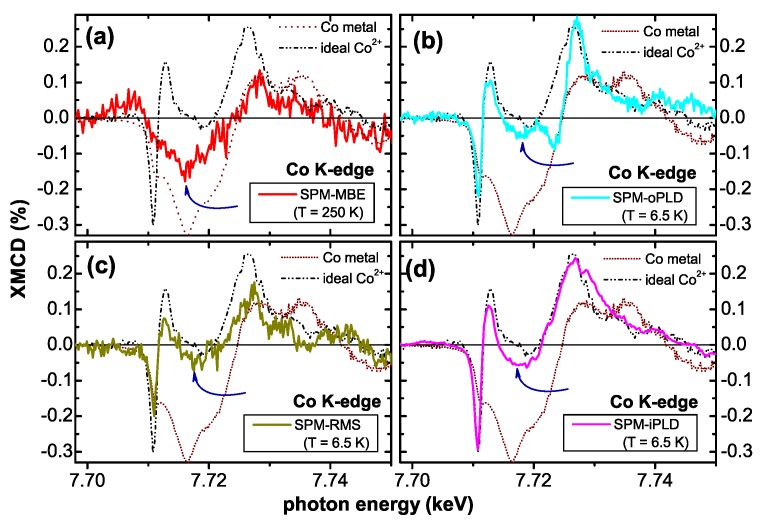
XMCD spectra recorded at the Co *K*-edge applying ±6 T in the film plane of (a) the SPM-MBE sample at 250 K. XMCD spectra recorded at 6.5 K are shown for (b) the SPM-oPLD, (c) SPM-RMS, and (d) of the SPM-iPLD. In all four panels reference XMCD spectra are shown for Co metal and ideal Co${}^{2+}$ (the PM-oPLD). The arrow marks the maximum of the XMCD of Co metal.

Figure 12 displays the respective XMCD spectra of all four SPM samples recorded at the Co *K*-edge. Whereas the XMCD of the SPM-MBE sample in Figure 12 (a) was recorded at 250 K to suppress PM contributions, the three other spectra were taken at 6.5 K. Figure 12 also includes two reference spectra from a metallic Co foil and from the PM-oPLD sample which can be considered as being representative for Co ideally incorporated on Zn lattice sites in ZnO (termed “ideal Co${}^{2+}$" in the following). Two characteristic spectral features can be seen: (i) the XMCD at the pre-edge feature is reduced; since it is not present in metallic Co it is indicative of the PM response of ideal Co${}^{2+}$. (ii) All SPM samples exhibit non-zero XMCD at photon energies where metallic Co exhibits a maximum and ideal Co${}^{2+}$ has zero XMCD signal. This can be taken as first indication that the SPM properties in all samples may be related to a metallic/elemental Co species. A closer inspection of Figure 12 reveals, that XMCD of the SPM-RMS (c) and SPM-iPLD (d) can be composed of a superposition of the ideal Co${}^{2+}$ and the metallic Co spectra, while the XMCD of the SPM-oPLD (b) has a different fine structure indicative of an additional Co species.

**Figure 13:**
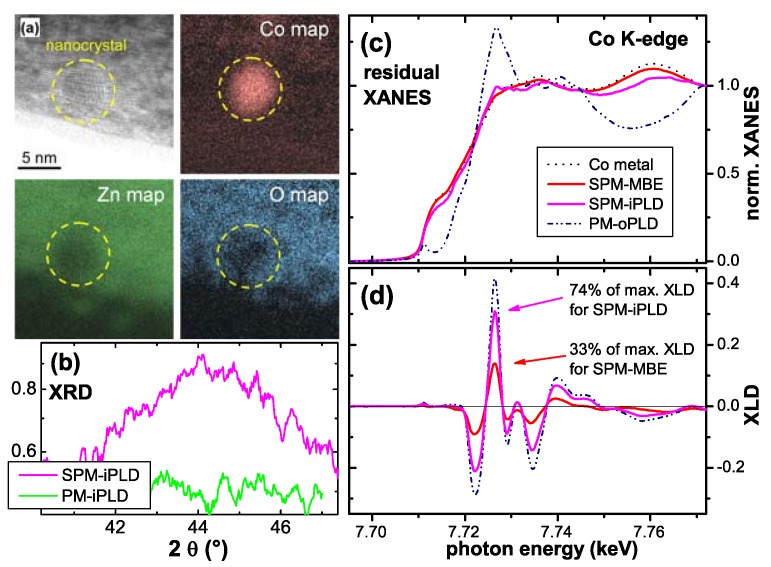
(a) Metallic Co nanoclusters in the SPM-iPLD sample: High-resolution TEM image revealing a nanocrystal with a diameter of about 4 nm. This region is rich in Co, but deficient in Zn and O demonstrated by energy filtered TEM. (b) *ω*-2*θ* XRD scans revealing an additional reflection for the SPM-iPLD sample which is absent for the PM-iPLD sample. (c) Residual XANES signatures at the Co *K*-edge of the SPM-MBE and SPM-iPLD samples resembling the reference XANES of Co metal. The residual XANES was derived by subtracting the XANES of the PM-oPLD weighted by the relative reduction of the XLD which is shown in (d).

First, the SPM-MBE and the SPM-iPLD shall be discussed in more detail. The presence of metallic Co nanoclusters in the SPM-MBE has been established before [73], therefore Figure 13 (a) and (b) only focus on the SPM-iPLD. Figure 13 (a) shows four TEM images where the presence of small (approx. 4–5 nm in accordance with the Langevin analysis in Figure 11) phase separated clusters can be seen, which are Co-rich and Zn- and O-deficient as revealed by energy-filtering TEM. Figure 13 (b) shows complementary evidence for an additional crystallographic phase in the SPM-iPLD sample by means of XRD. By drastically increasing the integration time of an *ω*-2*θ*-scan, a broad reflection around $2\theta =44^{\circ }$ becomes visible, which is absent for the PM-iPLD sample (Figure 13 (b), green line). In this region, one expects reflections for fcc and hcp metallic Co as well as Co${}_{3}$O${}_{4}$ and ZnCo${}_{2}$O${}_{4}$ spinel. However, the large FWHM for this reflection precludes a positive identification of the phase separated compound and individual nanoclusters seen by TEM may not be representative for the entire sample either. Here, the XANES allows a more representative estimate of the phase separated Co species. Figure 13 (c) shows the residual XANES of the SPM-iPLD and the SPM-MBE samples associated with the phase separated Co species together with the reference XANES of Co metal and ideal Co${}^{2+}$. It is obvious that the residual XANES is virtually identical with the one from Co metal for both samples which demonstrates that the second Co species is metallic. The residual XANES in Figure 13 (c) was derived as follows: We use the ratio of the XLD signal for a given specimen to that of ideal Co${}^{2+}$ shown in Figure 13 (d). Then, the normalized XANES spectrum of the ideal Co${}^{2+}$ is multiplied by the XLD-ratio (0.74 for the SPM-iPLD and 0.33 for the SPM-MBE) and subtracted from the respective experimental XANES. The residual spectrum is then renormalized and contains the spectroscopic signatures of virtually all Co atoms which are not in the ideal Co${}^{2+}$ environment. Therefore it can be concluded, that the SPM-MBE sample contains 33% ideal Co${}^{2+}$ and 67% metallic Co and the SPM-iPLD contains 74% ideal Co${}^{2+}$ and 26% of metallic Co which is present in the form of nanoclusters as evidenced by TEM and XRD and indicated by SQUID and XMCD. It should be noted that only a combination of the full experimental tool-set is suitable to draw such a conclusion on solid grounds.

**Figure 14:**
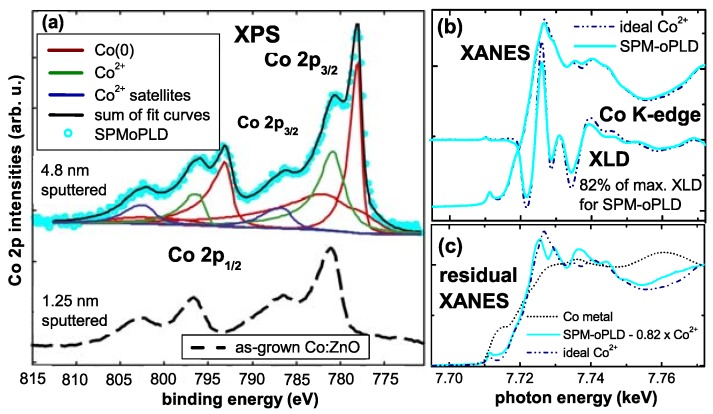
(a) Fit of an XPS spectrum of SPM-oPLD after sputter-removal of the topmost 4.5 nm of the film to a superposition of Co${}^{2+}$ and Co(0) revealing a fraction of metallic Co. For comparison an XPS spectrum for the as-grown sample after removal of the topmost 1.25 nm is shown. (b) XANES and XLD at the Co *K*-edge of the SPM-oPLD sample compared to the PM-oPLD sample. The residual XANES in (c) amplifies the spectroscopic signatures of the phase separated Co.

Figure 14 (a) shows an XPS spectrum of the SPM-oPLD sample recorded at the Co $2p_{3/2}$ and Co $2p_{1/2}$ emission lines in the vicinity of the surface of the film, *i.e.*, after removal of the topmost 4.5 nm by sputtering. Clear additional peaks at lower binding energies are visible, which are characteristic of elemental Co(0). The XPS spectrum is fitted to a superposition of Co${}^{2+}$ and Co(0) clearly indicating an increased fraction of metallic/elemental Co species close to the surface. The XPS depth-profile (not shown, see Reference [75]) demonstrates that the elemental Co(0) is located only near the surface of the film. The Co(0) enrichment at the surface originates from the Zn-diffusion of SPM-oPLD, since no signs of elemental Co(0) were found in the as-grown sample (Figure 14 (a), dashed line in the bottom). Note that a detailed EXAFS analysis indicated the formation of a CoZn intermetallic compound in a comparable sample (see References [75,76]). The XANES and XLD of the SPM-oPLD sample shown in Figure 14 (b) are very similar to ideal Co${}^{2+}$ underlining that only a small volume fraction of the sample is affected by the Zn-diffusion. The residual XANES in Figure 14 (c) exhibits an altrered fine structure and signs of increased elemental character at the pre-edge feature; both findings being in line with the formation of a ZnCo intermetallic compound.

**Figure 15:**
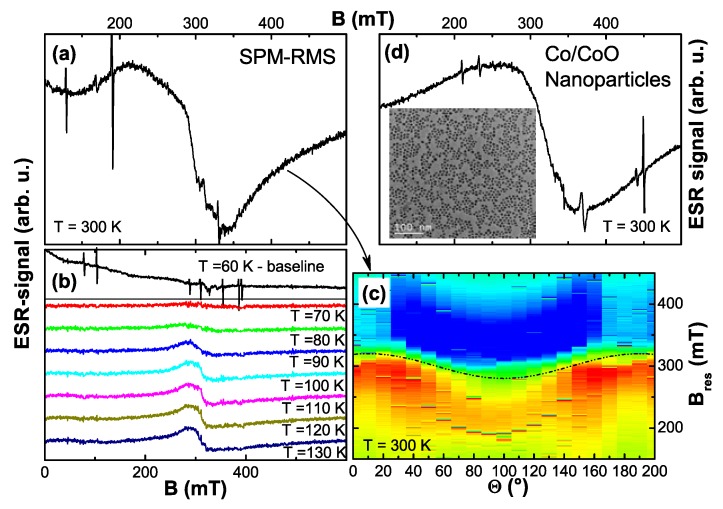
(a) EPR spectrum of the SPM-RMS sample recorded at 300 K. (b) shows the disappearance of the broad resonance line with decreasing temperature and (c) the polar angular dependence at 300 K as color contour plot revealing a weak uniaxial angular dependence (dash-dotted line). For comparison (d) displays the EPR spectrum of Co/CoO nanoparticles (inset: TEM image) recorded under identical conditions as (a).

In Figure 15 a different approach is shown to identify phase separation for the SPM-RMS sample by means of EPR at X-band frequencies. Figure 15 (a) displays a broad EPR resonance line recorded in out-of-plane geometry at 300 K, *i.e.*, at a temperature where the FC/ZFC curves already coincide according to Figure 11 (d), indicating the absence of magnetic hysteresis. In addition, the resonance is close to g=2, *i.e.*, despite the line being fairly broad, it is of PM nature. Note, that an EPR line of comparable shape has been taken as indication of FM elsewhere [68], whereas it was discussed in terms of phase separated Co clusters later on [146]. The broad EPR line is not observable at and below 60 K, see Figure 15 (b). Thus, this spectrum can be taken as baseline and subtracted from EPR spectra at more elevated temperatures. From Figure 15 (b) is can be seen that the EPR line starts to be visible around 80 K and its intensity increases with increasing temperature. Further, it exhibits a weak uniaxial anisotropy with the polar angle Θ which is shown at 300 K as color contour plot in Figure 15 (c). Such a weak anisotropy is known from Co nanoclusters [146] and can be well-explained by dipolar interactions. It exists a striking qualitative similarity of the EPR line of the SPM-RMS in Figure 15 (a) with the EPR response of Co/CoO nanoparticles recorded under the same experimental conditions as shown in Figure 15 (d). These nanoparticles were fabricated via a chemical route and are fairly monodisperse with an average diameter of ∼18 nm, see TEM image as inset in Figure 15 (d). Therefore, the origin of the EPR line in the SPM-RMS sample is a SPM resonance of the *unblocked* supermoments present in this sample, and therefore indicative of phase separation which could be corroborated by XRD and a reduced XLD as well [72]. A similar EPR line was also found in other SPM-RMS samples grown with further reduced oxygen partial pressure [147].

### 4.4. Magneto-transport Properties of PM/SPM Co:ZnO

For technological applications of DMS materials it is of secondary importance whether the magnetic response of Co:ZnO is of SPM or FM nature as long as large spin-dependent transport effects exist at and above room temperature. Some authors have demonstrated the presence of a small anomalous Hall effect (AHE) at room temperature in FM-like Co:ZnO [148]; however, in these samples soft-XMCD fails to corroborate the presence of FM [63]. Other authors report the presence of a measurable TMR signal in Co:ZnO; however these experiments were restricted to low temperatures [149]. It is therefore of interest, whether the SPM Co:ZnO with proven phase separation studied here exhibits characteristic magneto-transport signatures such as AHE or a hysteretic (“butterfly"-like) magneto-resistance (MR) at least at low temperatures.

**Figure 16:**
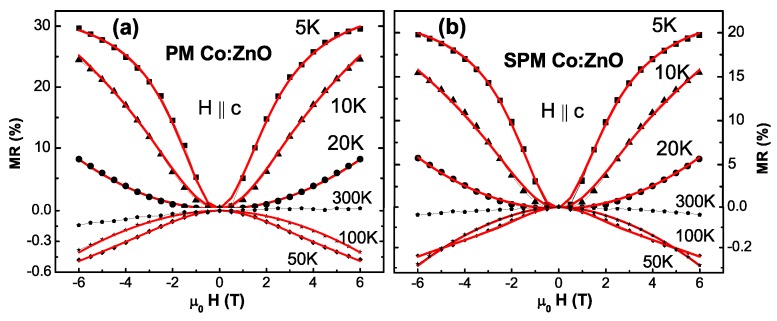
MR effect of a PM (a) and SPM (b) Co:ZnO sample with 5% of Co measured in van-der-Pauw geometry from 5 K to 300 K applying the magnetic field parallel to the *c*-axis. Red lines are fitting curves, see [145].

Figure 16 summarizes the MR effect as measured for a set of PM/SPM Co:ZnO samples containing 5% of Co. Besides the magnitude of the MR-effect at low temperatures, both films show qualitatively the same MR-behavior, indicating that the onset of phase separation in the SPM sample proven by a reduced XLD and an increased elemental character of the Co visible at the pre-edge feature of the XANES (not shown, see [145]) does not significantly alter the MR properties in these samples. In addition, no AHE was observed at any temperature. Only the *n*-type carrier concentration was altered from $2.4\times 10^{17}$/cm${}^{3}$ for the PM to $2.5\times 10^{19}$/cm${}^{3}$ for the SPM sample. In both cases the temperature dependence of the resistivity indicates hopping-type conductivity, presumably owing to the columnar growth which is typically found for epitaxial Co:ZnO films on *c*-plane sapphire. The transport and magneto-transport properties are discussed in more detail in [145] where also more details on the modeling of the MR-data (red lines in Figure 16) can be found. In brief, at more elevated temperatures the Co:ZnO films exhibit the usual negative MR known for undoped ZnO, e.g. [150] which is superimposed by a Brillouin-type positive MR of the paramagnetic Co-ions. These findings were recently corroborated for Co:ZnO fabricated by chemical decomposition of a precursor and dip-coating [151]. None of the observed magneto-transport properties could be attributed to the SPM behavior observed by integral and XMCD magnetometry in the RMS-grown samples.

### 4.5. Summary–Co:ZnO

Using a comprehensive set of complementary experimental techniques to characterize the structural and magnetic properties in an integral as well as element specific manner, we are able to demonstrate that phase pure Co:ZnO epitaxial films have the following *intrinsic* magnetic properties. (i) isolated Co${}^{2+}$ ions behave PM with a single ion anisotropy of the order of 3 K known from Co${}^{2+}$ impurities in bulk ZnO crystals. (ii) Neighboring Co dopant atoms forming Co-O-Co pairs couple antiferromagnetically. (iii) Any long range magnetic interaction beyond weak dipolar coupling evidenced by EPR-line-broadening could not be unambiguously identified. (iv) Slight deviations from the optimal growth conditions induce SPM which could be correlated with phase separation of Co-containing secondary phases. (v) XANES, XLD, and XMCD exhibit characteristic spectral features, which can be correlated with phase separation. (vi) No influence of the SPM phase on the magneto-transport properties could be identified.

Future experimental work aiming at manipulating the carrier concentration of Co:ZnO either by co-doping or other kind of defects has to exercise great care to rule out phase separation based on the preceding experimental methods. Without such insight based on a comprehensive set of experimental methods, establishing defensible cause-and-effect relationships between material properties and magnetism will not be possible.

## 5. Gd-doping of GaN Epitaxial Films

### 5.1. Fabrication of Gd-doped GaN

Two different types of Gd-doped GaN (Gd:GaN) samples will be discussed which were fabricated in two different institutions. Gd:GaN epitaxial films with a low nominal Gd-concentration (≤0.05%) have been grown using ammonia-assisted MBE directly on SiC(0001) substrates at the Paul-Drude Institut (PDI). Details of the sample characterization using *in situ* reflection high energy electron diffraction (RHEED), X-ray diffraction (XRD), and secondary ion mass spectroscopy (SIMS) can be found in References [36,95]. These samples originate from a growth series for which colossal magnetic moments have been claimed at low Gd concentrations of the order of 10${}^{16}$/cm${}^{3}$ [36]. They are typically highly resistive and contain about 10${}^{18}$/cm${}^{3}$ of oxygen. Higher nominal Gd concentrations were achieved by growing the Gd:GaN films on a MOCVD-grown GaN buffer on a Al${}_{2}$O${}_{3}$(0001) (*c*-plane sapphire) substrate by plasma-assisted MBE at the Georg-August Universität Göttingen (GAU), partially with Hydrogen co-doping. Also this growth method results in colossal magnetic moments at lower Gd concentrations as evidenced by SQUID [96]. In all cases the nominal Gd concentration was determined by extrapolation of the preparation conditions. Note, that a direct determination of the actual Gd concentration by SIMS has not been performed on the respective samples yet and may typically differ by up to a factor of 5. Gd:GaN samples fabricated by Gd-ion-implantation at the Ruhr-Universität Bochum were restricted to Gd concentrations below the detection limit of the synchrotron methods to avoid amorphization and will not be discussed here; results can be found in Refrences [98,99].

In the following, three different Gd:GaN samples will be discussed in more detail. One sample contains 0.05% of Gd and was grown at the PDI as one of a series of many Gd concentrations. The two other samples were fabricated at the GAU, one contains 2.9% of Gd and the other 1.9% of Gd. The latter was co-doped with H in addition.

### 5.2. Structural Properties

**Figure 17:**
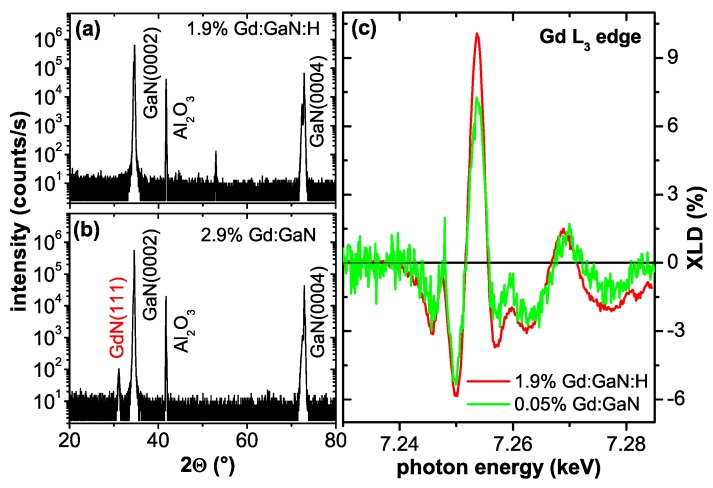
XRD *ω*-2*θ*-scans (a) for the 1.9% Gd:GaN sample co-doped with H with no signatures of phase separation and (b) for the 2.9% Gd:GaN sample clearly revealing a secondary phase identified as GdN. (c) XLD spectra recorded at the Gd $L_{3}$-edge of the 1.9% Gd:GaN:H (red) and the 0.05% Gd:GaN (green) samples.

Figure 17 compiles a summary of the structural characterization of the three Gd:GaN samples. The two samples fabricated at the GAU were studied by means of XRD. Whereas the 1.9% Gd:GaN:H sample shown in Figure 17 (a) does not show any signatures of phase separation in XRD *ω*-2*θ*-scans, the 2.9% Gd:GaN sample in (b) shows a clear additional reflection which can be attributed to GdN. In addition, the XLD at the Gd $L_{3}$-edge was recorded for all three samples. The XLD of the 2.9% Gd:GaN is discussed in more detail in [152]; the XLD of the 0.05% Gd:GaN sample in [113]. In Figure 17 (c) the XLD of the 1.9% Gd:GaN:H sample is displayed together with the one of the 0.05% Gd:GaN sample. For the latter, FDMNES simulations have indicated that ∼15% of the Gd is not located on substitutional sites [113]. Accordingly, the XLD is smaller than for the 1.9% Gd:GaN:H sample pointing towards a better incorporation of the Gd into the GaN lattice highlighting the beneficial influence of H co-doping. Note, that virtually all spectroscopic signatures in the XLD can be seen in both spectra indicating the limitations of the respective XLD simulations in [113] which cannot reproduce all the subtleties of the experimental XLD. This can be attributed to measuring/simulating *L*-edges, where the final states have *d*-character and thus they are more localized than the *p*-states probed at the *K*-edges which extend further out into the crystal field. Consequently, the XLD at *L*-edges is typically a factor of 10 smaller than at *K*-edges and thus more difficult to simulate.

### 5.3. Magnetic Properties

So far, the three samples can be classified as follows: The 1.9% Gd:GaN:H sample seems to contain little if any secondary phases. For the 2.9% Gd:GaN phase separation of GdN has been proven. For the 0.05% Gd:GaN sample about 85% of the Gd are well-incorporated into the GaN lattice but phase separation may be present. In the following these structural properties will be linked with the respective integral and element specific magnetic properties.

#### 5.3.1. Integral Magnetometry

**Figure 18:**
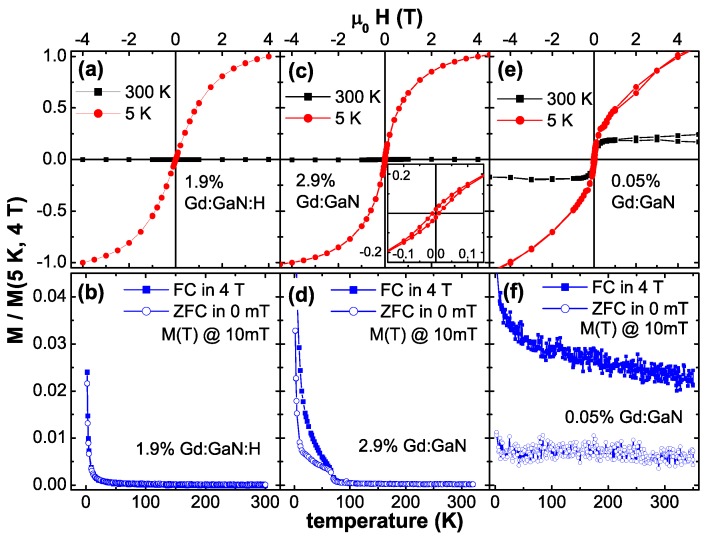
SQUID measurements of the three Gd:GaN samples. (a) displays M(H)-curves at 300 K and 5 K and (b) the respective M(T) in FC and ZFC conditions of the 1.9% Gd:GaN sample revealing PM. (c) and (d) display the respective data of the 2.9% Gd:GaN sample; the inset in (c) enlarges the low field regime. (e) and (f) collate the SQUID data of the 0.05% Gd:GaN sample. All data are normalized to M(5 K, 4 T) and the diamagnetic background of the substrate has been derived from the high-field behavior of the 300 K data and subtracted from all data sets.

Figure 18 summarizes the findings of integral SQUID magnetometry for all three Gd:GaN samples. The M(H)-curves at 300 K and 5 K of the 1.9% Gd:GaN:H sample in Figure 18 (a) and the respective M(T)-curves under FC and ZFC conditions (b) reveal virtually pure PM corroborating the phase pureness of this sample. Note that a closer inspection of the shape of the M(H)-curve reveals minor deviations from the expectations of a Brillouin function $B_{J}$ for J=S=7/2; however, these will not be discussed here. In contrast, the respective M(H) and M(T)-curves in Figure 18 (c) and (d) of the 2.9% Gd:GaN sample exhibit a clear hysteretic behavior at 5 K which is consistent with a FC-ZFC splitting at temperatures below ∼70 K, which is a characteristic Curie temperature for GdN, see e. g., [153]. Thus the SQUID data are indicative of SPM behavior which can be attributed to the phase separation of the GdN evidenced by SQUID. The SQUID data of the 0.05% Gd:GaN sample in Figure 18 (e) and (f) are in stark contrast to the other Gd:GaN samples. While at low temperatures a PM component is visible, the magnetic behavior at more elevated temperatures is dominated by a strong FM-like behavior. An additional SPM contribution below ∼70 K is barely visible but known from similar Gd:GaN fabricated at the PDI [154]. For technological applications only the FM-like contribution is of interest and would constitute useful evidence of a DMS material with magnetic order at RT. However, this result requires confirmation by a second, complementary experiment which will be tried in the following.

**Figure 19:**
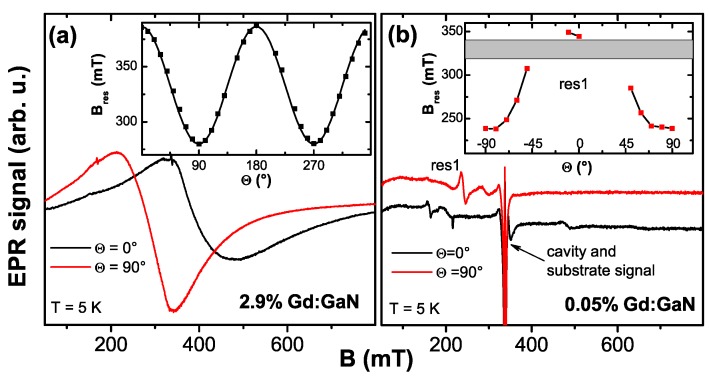
EPR measurements as a function of the polar angle Θ at X-band frequency and 5 K for the (a) 2.9% and (b) 0.05% Gd samples, respectively. (a) A broad resonance line with uniaxial angular dependence (see inset) is visible for the 2.9% Gd sample. (b) The 0.05% Gd sample shows various weak signals; the most prominent line (labeled “res1") shows a uniaxial behavior as well (see inset).

The EPR investigations using X-band frequency are summarized in Figure 19. The 2.9% Gd:GaN sample containing GdN nanoclusters is shown in Figure 5 (a) and it exhibits a very broad and asymmetric resonance line which shows a uniaxial angular dependence around g=2 at 5 K as shown in the inset. These features—already discussed along with Figure 15—are characteristic for a blocked superparamagnetic ensemble. The temperature dependence is consistent with the FC/ZFC measurements by SQUID in Figure 18 (d). The EPR spectra of 0.05% Gd:GaN sample shown in Figure 19 (b) are dominated by a strong background around g=2 stemming from the substrate and the microwave cavity as discussed in more detail in Reference [155]. Most remarkable, no EPR resonance, which can explain the strong SQUID response in Figure 18 (e) and (f), is observed. The most prominent EPR line at 5 K, which is much weaker and more narrow compared to the 2.9% Gd sample, shows a uniaxial behavior. This line vanishes quickly with temperature (not shown, see [152,155]). The EPR findings in this sample are best explained with isolated, non-interacting Gd or GdN clusters and a comparison with a spin-reference yields about 15% of the Gd contributing to this resonance [155]. This fraction is in good agreement with the amount of non-substitutional Gd evidenced by XLD in Figure 17 (c) and [113]. The ferromagnetic-like character of this line can be taken as indication of the onset of phase separation. Signatures of isolated, paramagnetic Gd impurities are not visible, in particular, because the respective resonance field is presumably covered by the cavity and substrate signals. To shed further light on the magnetic properties, the Gd sublattice magnetization will be probed via XMCD in the following.

#### 5.3.2. Gd Sublattice Magnetization

**Figure 20:**
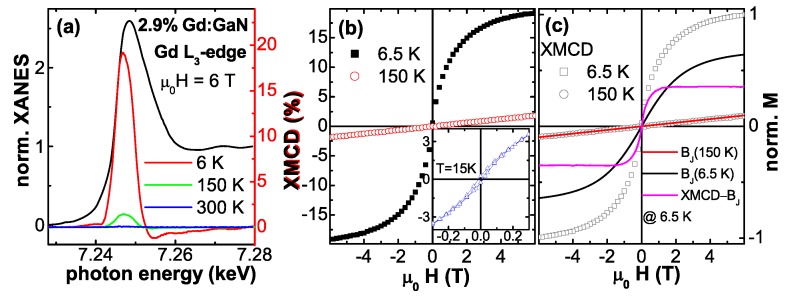
(a) XANES and XMCD recorded at the Gd $L_{3}$-edge of the 2.9% Gd:GaN sample at 6 T and various temperatures. (b) M(H)-curves recorded at the Gd $L_{3}$-edge at 6.5 K (black squares) and 150 K (red circles). The the inset reveals weak hysteretic behavior at 15 K. (c) Modeling of the XMCD M(H)-curves (gray symbols) using the Brillouin function $B_{J}$ (see text).

The XMCD spectra recorded in 6 T at 6 K, 150 K and 300 K at the Gd $L_{3}$-edge are shown in Figure 20 (a) for the 2.9% Gd:GaN sample. The maximum XMCD signal at 6 K is about 20% which can be correlated to a magnetic moment exceeding the atomic moment of 8 $\mu_{B}$/Gd by comparison with published data for GdN [153]. The XMCD is strongly reduced at 150 K and is not observable any more at 300 K. In Figure 20 (b) the M(H)-curves recorded at 6 K and 150 K are shown revealing PM-like behavior; however, the inset in Figure 20 (b) reveals that a small magnetic hysteresis opens up at 15 K which is not observable at 100 K any more (not shown). In Figure 20 (c) two different contributions to the M(H)-curves at 6 K are disentangled by the following procedure: Since at 150 K the sample is PM according to the SQUID results in Figure 18 (c) and (d), an atomic-like magnetic moment is expected for all Gd atoms. Thus, the M(H)-curve at 150 K is modeled using a Brillouin function $B_{J}$ using J=S=7/2 and adjusted to the experimental XMCD M(H) data (red line). Then the expected PM response at 6 K can be calculated which is shown as black line in Figure 20 (c). The additional magnetic contribution can then be estimated by subtracting a weighted $B_{J=7/2}(6$ K) from the experimental M(H)-curve. This difference is plotted as purple line for $0.7\times B_{J}$ in Figure 20 (c). It is obvious that about 30% of the Gd exhibit an additional magnetic response which can be described by a Langevin (*L*) function with an effective magnetic moment *μ* ∼50 $\mu_{B}$ which can be assigned to the SPM behavior of the phase separated GdN. Most of these GdN clusters are unblocked at this temperature as indicated by the small hysteretic contribution to the overall magnetic response visible in Figure 18 (c) and Figure 20 (b). Note that the preceding analysis is only meaningful for element-specific M(H)-curves.

**Figure 21:**
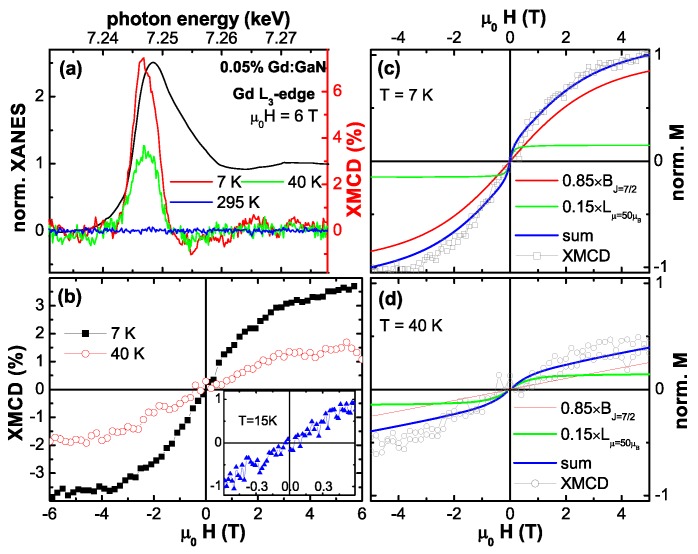
(a) XANES and XMCD recorded at the Gd $L_{3}$-edge of the 0.05% Gd:GaN sample at 6 T and various temperatures. (b) M(H)-curves recorded at the Gd $L_{3}$-edge at 7 K (black squares) and 40 K (red circles). The inset reveals anhysteretic behavior at 15 K. Modeling of the XMCD M(H)-curves (gray symbols) using a superposition of a Brillouin ($B_{J}$) and a Langevin (*L*) function at (c) 7 K and (d) 40 K (see text).

The XMCD spectra at the Gd $L_{3}$-edge for the 0.05% Gd:GaN sample are shown in Figure 21 (a) which were recorded in 6 T at 7 K, 40 K and 295 K. The maximum XMCD signal at 7 K is only about 7% which is approximately equal to the atomic moment of 8 $\mu_{B}$/Gd as discussed before [113]. The XMCD reduces at 40 K and is not observable at 295 K. Thus, the Gd sublattice cannot be responsible for the SQUID signal in Figure 8 (e) and (f) at more elevated temperatures. In Figure 21 (b) the respective M(H)-curves are shown, revealing anhysteretic PM-like behavior which is corroborated by the data shown in the inset, where the low-field regime was measured at 15 K. The Gd sublattice M(H) behavior is obviously clearly distinct from the integral magnetization measurements by SQUID. Since the XLD in Figure 17 (c) and the EPR data in Figure 19 (b) have already provided evidence of about 15% of the Gd being present as clusters, the M(H)-curves of the Gd sublattice are modeled in Figure 21 at 7 K (c) and 40 K (d). The Gd sublattice M(H)-curves cannot be fitted well by using a single Brillouin function [113]. Like for the 2.9% Gd:GaN sample the data are fitted by a superposition of $B_{J}$ with J=7/2 and *L* with *μ* = 50 $\mu_{B}$, where the best fit is achieved for 85% of the atomic-like $B_{J}$ contribution and 15% of Gd being present as SPM clusters consistent with the XLD and EPR results.

#### 5.3.3. Magnetic Polarization of the GaN Host

So far, it has been demonstrated that the Gd sublattice of the 0.05% Gd:GaN behaves essentially PM, with a small (15%) fraction of the Gd being present as SPM clusters. Thus, the SQUID data comprise an additional magnetic component at more elevated temperatures. In the following the origin of this magnetic contribution shall be further investigated. For this two different approaches are chosen: (i) magneto-photoluminescence (PL) measurements and (ii) an XMCD study at the Ga *K*-edge to clarify whether the additional magnetic signal is caused by the GaN host crystal itself.

**Figure 22:**
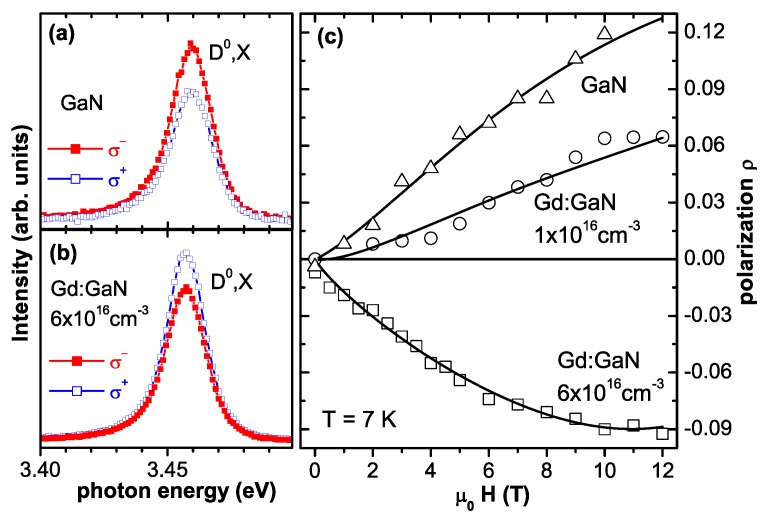
Circularly polarized photoluminescence spectra of the donor-bound exciton ($D^{0},X$) for (a) a undoped GaN reference sample and (b) an GaN sample doped with $6\times 10^{16}$/cm${}^{3}$ Gd. Both samples were measured at 7 K and 10 T in the Faraday configuration (B∥c). (c) Circular polarization *ρ* of the ($D^{0},X$) emission as a function of the external magnetic field at 7 K for the reference GaN sample (triangles) and two Gd:GaN films with $1\times 10^{16}$/cm${}^{3}$ of Gd (circles) and the sample in (b) (squares). Data are from [156].

First, the band structure of the Gd:GaN sample shall be probed by magneto-PL. The PL spectra of this series of Gd:GaN samples are dominated by the donor-bound exciton ($D^{0},X$) transition at a photon energy of 3.458 eV. The donor most likely being responsible for this transition is oxygen with a concentration of about 10${}^{18}$/cm${}^{3}$ as measured by SIMS. Since these donors are distributed homogeneously over the entire GaN matrix, the properties of the electronic band structure can be probed. Figure 22 shows the PL spectra at 10 T and 7 K for an undoped GaN reference sample (a) and a $6\times 10^{16}$/cm${}^{3}$ Gd:GaN sample (b) stemming from the same growth series as the 0.05% Gd:GaN sample. The observed ($D^{0},X$) emission is polarized in both samples, which is evident from the difference in intensities of the two circularly polarized $\sigma^{-}$ (full squares) and $\sigma^{+}$ (open squares) components. Most importantly, the polarization for Gd:GaN sample has the opposite sign of the one in the GaN reference sample. Figure 22 (c) displays the field dependence of the ($D^{0},X$) polarization *ρ* for the two samples in (a) and (b) and an intermediate Gd concentration. All data in Figure 22 can be found in [156]. Beyond the discussion in terms of magnetic interactions in [156] for the purpose of the present paper the field dependence is of interest. It is clear from Figure 22 (c) that even at very low temperatures the magnetic polarization at remanence is rather small if present at all. Further, the polarization decreases quickly with increasing temperature (not shown, see [156]). Therefore, the magnetic properties as seen by the ($D^{0},X$) transition, *i.e.*, the band structure, presumably influenced by the presence of the oxygen, are of PM character and therefore cannot account for the M(H) and M(T) behavior at 300 K as seen in Figure 18 (e) and (f).

**Figure 23:**
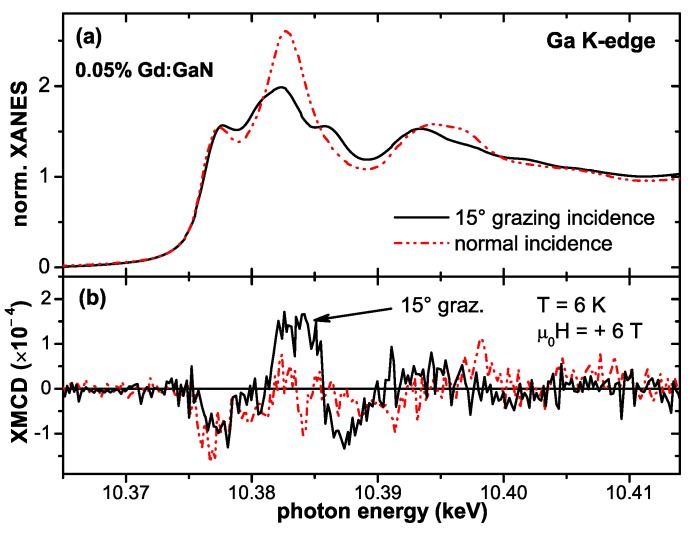
(a) Normalized XANES spectra at the Ga *K*-edge of the 0.05% Gd:GaN sample recorded at 6 K for 15${}^{\circ }$ grazing incidence (full black line) and normal incidence (dash-dotted red line) of the X-rays. (b) Respective normalized XMCD signal at 6 T for both orientations.

Second, the GaN matrix is directly probed by XMCD. Figure 23 (a) shows XANES spectra at the Ga *K*-edge for 0.05% Gd:GaN sample recorded at 7 K with the X-ray beam at normal incidence and 15${}^{\circ }$ grazing incidence. A comparison of the XANES spectra for both geometries shows clear differences in the fine structure. In the case of grazing incidence the X-ray *E* vector rotates in the *a*-*c*-plane whereas under normal incidence it remains within the *a*-plane. The difference in the XANES spectra is therefore indicative of the presence of a substantial XLD effect as measured for the *c*-oriented wurtzite structure of GaN as seen in Figure 1. Figure 23 (b) shows the respective XMCD spectra which were recorded at 7 K in an external field of 6 T for grazing and normal incidence. The XMCD at the Ga *K*-edge is a measure of only the 4p orbital contribution to the total magnetic moment. We can detect an XMCD signal of the order of 0.013% at the Ga *K*-edge for 15${}^{\circ }$ grazing and normal incidence exhibiting relatively similar spectral features. From SQUID measurements an overall magnetic polarization of $1.1\times 10^{-3}$$\mu_{B}$ has been inferred inside the “spheres of influence" [36]. For a quantitative comparison, the size of the magnetic moment of the Ga has to be estimated via the XMCD at the Ga *K*-edge. The XMCD results in Figure 23 (b) can be compared to data recorded at the Ga *K*-edge of (InGaMn)As [157]. Here a Ga 4p orbital moment of $8\left(4\right)\times 10^{-5}\mu_{B}$ has been correlated with a maximum XMCD intensity of 0.05%. In turn this means that from the size of the XMCD in Figure 23 (b) one can roughly estimate a magnetic moment of the order of at most $10^{-5}\mu_{B}$ as *upper* bound, since the integral of the XMCD in Figure 23 (b) is close to zero, whereas in Reference [157] the spectral shape has only one positive feature, *i. e.*, a much larger integral. This estimate is between one and two orders of magnitude smaller than what is expected from the empirical model. Therefore, the magnetic signal recorded by integral SQUID magnetometry cannot be attributed to the magnetic polarization of the Ga. Since the inferred sphere of influence shall extend over about 28 nm [36], it is furthermore rather unlikely that the polarization is only carried by the N anions without polarizing the Ga cation in-between. Nonetheless, no direct XMCD measurement has been performed at the N *K*-edge so far.

### 5.4. Summary–Gd:GaN

The detailed analysis of the element specific and integral magnetic properties of Gd:GaN DMS samples reveals the following: (i) The magnetic order found in 0.05% Gd:GaN at room temperature by SQUID measurements cannot be corroborated by complementary experimental techniques. (ii) Phase separated Gd or GdN clusters leading to a blocked SPM order below 70 K are found for 2.9% Gd:GaN. (iii) Phase separation of Gd or GdN clusters can be suppressed by co-doping with H leading to a PM behavior of a 1.9% Gd:GaN:H sample. (iv) In the 0.05% Gd:GaN sample signatures of the onset of phase separation are already visible by means of EPR and M(H)-curves by XMCD. (v) The magnetic polarization of the Ga sites in the 0.05% Gd:GaN sample is by two orders of magnitude too small to account for the colossal effective magnetic moments; the magneto-PL of the $D^{0},X$ emission is only indicative of PM as well.

The magnetic order at room temperature at very low Gd concentrations of Gd:GaN DMS materials could not be corroborated by complementary experimental techniques. The exceptional magnetic properties of Gd:GaN as measured by integral SQUID magnetometry can neither be assigned to the Ga or the Gd sites as probed by element specific synchrotron measurements nor can EPR provide supporting experimental evidence. Characteristic signatures of phase separation are found at high concentrations by means of EPR, XLD, and element specific M(H)-curves which can be suppressed by H co-doping. This comprehensive tool-box shall enable to establish or disprove the existence of RT FM in Gd:GaN in future experiments on more solid grounds beyond SQUID magnetometry.

## 6. Conclusions and Outlook

In conclusion, two wide band-gap DMS materials, Co:ZnO and Gd:GaN, have been studied by hard X-ray absorption spectroscopy, in particular XLD, XMCD and element specific M(H)-curves. These findings have been compared with results from integral SQUID magnetometry as well as EPR. For both DMS materials room temperature FM could not be confirmed. However, signs of phase separation and clustering of the respective dopant species are found in all samples which exhibit SPM behavior therefore inferring an extrinsic origin of the magnetic properties of the DMS material which does not affect the transport properties of SPM Co:ZnO. Complementary magnetometry such as EPR or synchrotron-based methods have been shown to be virtually indispensable to establish defensible cause-and-effect relationships between material properties and magnetism. The use of the structural XLD can provide quantitative information about the incorporation of the dopant into the host lattice in addition. Such an experimental approach is of significant potential value to a wide range of researchers investigating dilute systems or other complex materials in general. In particular, it has the potential to settle the controversy about Co:ZnO and provides opportunities to unravel the origin of the colossal magnetic moments reported for Gd:GaN. Finally, it should have become obvious that the presence of a magnetic hysteresis in SQUID experiments is only a necessary but not a sufficient criterion to claim the existence of FM in DMS materials. The present findings may also explain why no spintronic device based on DMS materials which operates at room temperature has been successfully demonstrated yet. Therefore, the challenge remains open for material-scientists world-wide to improve the performance of integrated circuits by combining the best of two worlds, semiconductors and ferromagnetism, for non-volatile, reprogrammable, and power-efficient computing.
